# Role of tectonic stress and topography on repeated lateral dikes: application to the 1975–1984 Krafla and 2023–2025 Svartsengi rifting episodes in Iceland

**DOI:** 10.1007/s00445-025-01897-y

**Published:** 2025-11-04

**Authors:** Yilin Yang, Freysteinn Sigmundsson, Halldór Geirsson, Joachim Gottsmann

**Affiliations:** 1 Nordic Volcanological Center, Institute and Faculty of Earth Sciences, University of Iceland, Reykjavík, Iceland; 2 Department of Earth and Environmental Sciences, Ludwig‐Maximilians‐Universität München, Munich, Germany

**Keywords:** Repeated lateral dikes, Krafla rifting episode, Svartsengi rifting episode, Iceland, Magma overpressure, Tectonic stress, Topography

## Abstract

Lateral dike intrusions into rift zones, repeated in temporal sequences, depend on intrinsic and extrinsic factors including pressure build-up where magma accumulates between intrusions, and the local stress regime influenced by tectonic stress and topography. We reexamine these effects by revising a simplified elastic model of rifting by Buck et al. (2006), where topographic gradients and tectonic stress, in addition to magma accumulation, contribute to the driving pressure of dike propagation. Our model (i) introduces a three-section dike geometry, (ii) assumes lateral dike intrusions follow a positive pressure gradient and open in the part of the rift zone where the local maximum of driving pressure occurs, (iii) restricts the amount of dike opening according to tectonic stress stored prior to a rifting episode, and (iv) incorporates magma compressibility to better understand volume change in a magma domain. As successive intrusions redistribute stress, dike opening is favored at different parts along the rift zone. When applied to the 1975–1984 Krafla rifting episode, the model predicts a spatial distribution of dike openings consistent with geophysical observations, with magma flow into the Krafla fissure swarm through an inlet located ~ 2–4 km north of the Krafla caldera center. Inferred magma pressure at the inlet at the initiation of the first dike is ~ 1–10 MPa above lithostatic stress, whereas tectonic stress and topographic effects contributed > 20 MPa to the driving pressure. The failure pressure limit for the first dike is found to be larger than that for later dikes by a factor of 2. The lower failure limit for later dikes and tectonic stress allow magma flow into subsequent dikes when magma pressure at the inlet is below lithostatic. The model is also applied to fit the spatial distribution of dike openings in the beginning of the 2023–2025 Svartsengi rifting episode in SW Iceland. There, topographic effects contribute ~ 2 MPa of driving pressure to the southern propagation of the first dike. Tectonic stress and significant magma buoyancy (up to 9 MPa) enable dike initiations when the overpressure from magma recharge in the domain was below lithostatic. Tectonic stress inhibited eruption from the initial dike of the Svartsengi rifting episode. Our findings demonstrate that tectonic stress and topographic effects are critical factors driving lateral dike propagation, allowing magma flow into dikes or eruptions under low magma pressure, even lower than lithostatic pressure.

**Supplementary information:**

The online version contains supplementary material available at 10.1007/s00445-025-01897-y.

## Introduction

Dike intrusion is a fundamental process transporting magma vertically and laterally in the Earth’s crust (e.g., Dahm 2002; Daniels and Menand 2015; Urbani et al. 2018). At extensional plate boundaries, repeated lateral dike intrusions often occur in rifting episodes lasting from months to years, with dikes propagating up to tens of kilometers along rift zones (fissure swarms in Icelandic terminology; e.g., Jóhannesson and Sæmundsson 2009; Hjartardóttir et al. 2012, 2015). Temporal sequences of dikes during rifting episodes release stress accumulated in plate boundary deformation zones over large timescales (centuries) due to plate movements (e.g., Abdallah et al. 1979; Paquet et al. 2007; Wright et al. 2012). Such dikes can cause meter-scale ground deformation, trigger normal faulting, and lead to eruptions (e.g., Björnsson et al. 1977; Biggs et al. 2009; Grandin et al. 2010; Parks et al. 2023; Sigmundsson et al. 2024). Studying their spatial features and controlling factors enhances our understanding of divergent plate tectonics, magma transport, and eruptions and assists in volcanic hazard assessment.

### Dike models

Dike propagation models primarily aim to predict the temporal evolution of a single dike, including its geometry, propagation velocity, and magma pressure distribution. Traditionally, there have been two main schools of thought for modeling the vertical propagation of a single dike (Nakashima 1993; Rivalta et al. 2015). One emphasizes the resistance of host rock to fracturing, deriving dike geometry from the minimum fluid pressure to maintain equilibrium of an open fluid-filled crack. The other emphasizes the stress in viscous magma flow to predict dike propagation dynamics. Hybrid models incorporating assumptions from both schools of thought have been developed for complex scenarios (Dahm et al. 2000; Roper and Lister 2007), with some extending to lateral dike propagation. For instance, Rivalta (2010) considered lateral dikes and a magma reservoir coupled by a conduit with viscous magma flow to model the temporal evolution of dike propagation. Townsend et al. (2017) introduced a third school of thought, focusing on the vertical cross-sections of lateral dikes, and their propagating distance and horizontal extent. However, reproducing the spatial features of temporal dike sequences and how they release accumulated stress at divergent plate boundaries require a different modeling approach, which quantifies tectonic stress evolution along the rift zone as dikes intrude.

A simplified numerical model of dike sequence formation was presented by Buck et al. (2006). The model analyzed tectonic and topographic stress along the rift zone and predicted major dike openings by assuming that their associated stress changes are controlled by the difference between magma pressure and stress conditions along the fissure swarm due to tectonic stress and topographic effects. When applied to the 1975–1984 Krafla rifting episode in Iceland, the model predicted dike opening distribution broadly consistent with field observations. However, there is scope for improvement. For example, the model assumes a uniform stress distribution along the dike at the end of an intrusion, with dike opening that can be larger than expected from accumulated tensile stress due to plate spreading prior to a rifting episode. Observations show, however, that a single dike in a rifting episode may only partially release previously accumulated tectonic stress, and the total stress release of a dike sequence may be limited by the magnitude of tectonic stress accumulated prior to a rifting episode (e.g., Wright et al. 2012; Heimisson et al. 2015b; Parks et al. 2023; Sigmundsson et al. 2024; Greiner et al. 2025). Additionally, the Buck et al. (2006) model assumed a vertical dike extent of 10 km at Krafla, whereas geodetic observations suggest a range of 2–5 km (Hollingsworth et al. 2013). Modifying the assumed dike geometry and how the model releases tectonic stress in each dike intrusion can provide further insights into the factors controlling dike sequence behavior during rifting episodes.

### Krafla volcano and the 1975–1984 rifting episode

**Fig. 1:**
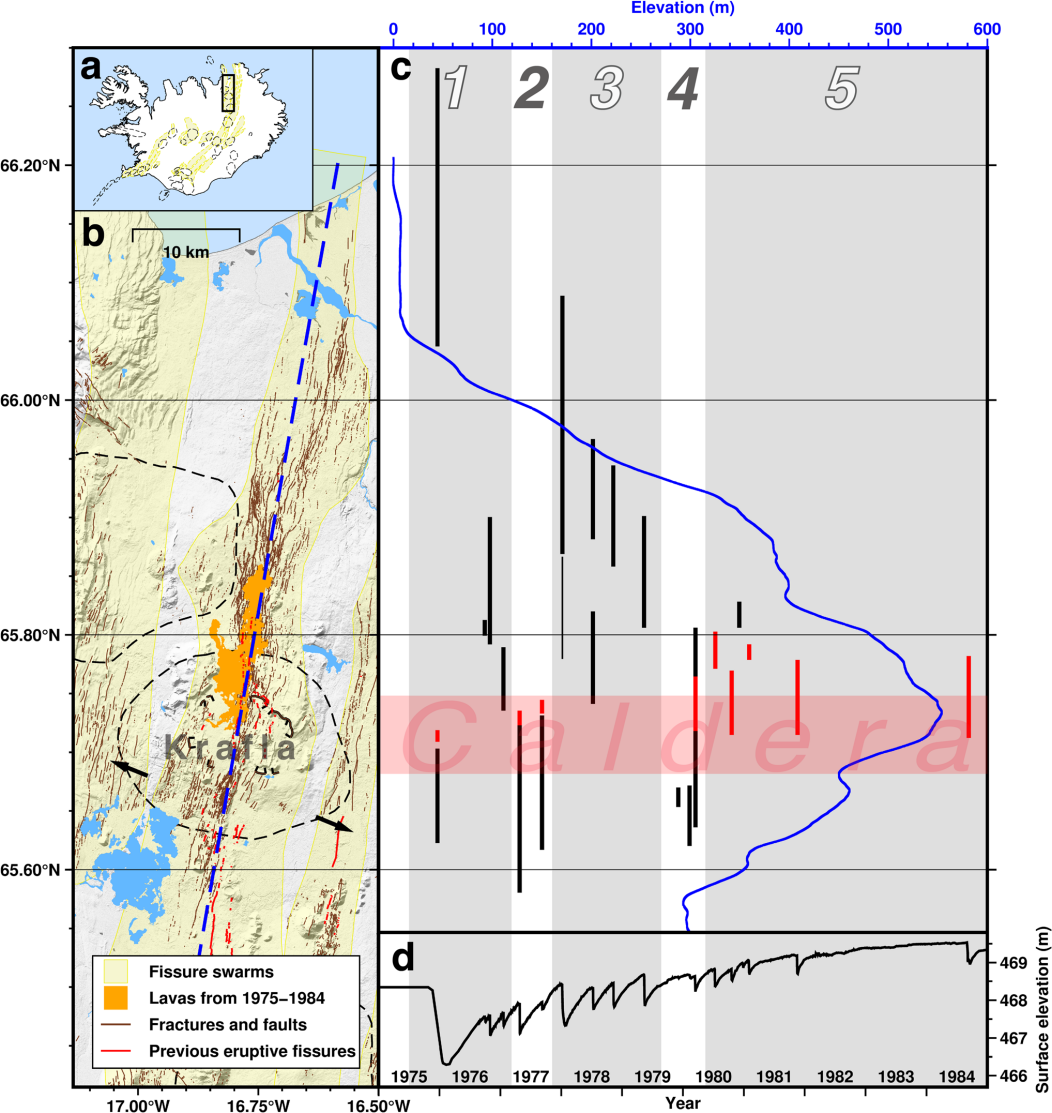
Krafla volcano and the dikes formed in the 1975–1984 rifting episode. **a** A map of Iceland showing the location of the Krafla volcanic system (black rectangle). **b** A map of the Krafla volcanic system. The black dashed lines indicate central volcanoes and tick-marked lines indicate the outline of the Krafla caldera where mapped. Blue dashed line shows the location of the elevation profile shown in Fig. 1b. The arrows indicate the direction of plate spreading. The topography is from LMÍ (2020). Fractures are from Hjartardóttir et al. (2015). The Krafla fissure swarm, central volcano, and caldera are from Sæmundsson (1991). **c** Elevation profile along the Krafla fissure swarm smoothed with Savitzky-Golay filter (blue line), and the distance range in the Krafla fissure swarm with main dike opening (black) indicated by seismic swarms and eruptions (red), adapted from Einarsson (1991) and Buck et al. (2006). The horizontal red shaded area indicates the latitude range of the Krafla caldera, and the vertical gray and white straps indicate the different sub-sequences of dikes numbered on the top. **d** Elevation change in the Krafla caldera center during 1975–1984 Krafla rifting episode, from Björnsson and Eysteinsson (1998)

The Krafla volcano is in the Northern Volcanic Zone (NVZ) of Iceland, at the divergent Eurasian North-American plate boundary (Fig. 1). Its volcanic system comprises a central volcano with a diameter of ~ 25 km and a 5–8 km wide transecting fissure swarm extending approximately 40 km to the south and 50 km to the north (Opheim and Gudmundsson 1989; Hjartardóttir et al. 2012; Lanzi et al. 2023). A 9 × 7 km caldera formed ~ 100,000 years ago at the central volcano (Sæmundsson 1991). The average plate spreading rate is 17.4 mm/yr in direction N112° E in this area (DeMets et al. 2010; Drouin et al. 2017). Drilling, seismological, and petrological studies suggest the presence of magma across a wide range of depth beneath the caldera (2.1–19 km; e.g., Mortenson et al. 2014; Einarsson 1978; Tryggavson 1980, Brandsdóttir et al. 1997; Schuler et al. 2015; Kim et al. 2020; Rooyakkers et al. 2024).

The most recent rifting episode at Krafla in 1975–1984 consisted of about 20 rifting events with dike intrusions affecting a portion of the fissure swarm, nine of which led to eruptions (e.g., Björnsson et al. 1979; Tryggvason 1980; Einarsson 1991; Wright et al. 2012). These events generated seismic swarms, faulting, and surface widening. The cumulative surface widening averaged 4–5 m, corresponding to 200–250 years of accumulated spreading, with the maximum cumulative widening up to ~ 8 m north of the Krafla caldera (Tryggvason 1984; Hollingsworth et al. 2012). Geodetic data revealed gradual inflation in the caldera between diking events, followed by sudden deflation beginning at the onset of each dike intrusion (Fig. 1d). The subsidence reached > 1.8 m during the first dike in 1975, which recovered only ~ 40% by inflation before the second dike initiated. Thereafter, the elevation at dike onsets gradually increased and had recovered to the level prior to the rifting episode at the onset of the 1979–1980 events (Tryggvason 1980; Johnsen et al. 1980; Einarsson 1991; Buck et al. 2006). Hollingsworth et al. (2013) derived ground displacements along the Krafla fissure swarm during the rifting episode from aerial photos and inferred the dikes extending from 0–3 to 5 km depth in the crust. Geodetic models constrained by leveling and distance measurements, using a point pressure source in a homogeneous elastic half-space (Mogi 1958), estimated the source depth at ~ 3–4.3 km (e.g., Tryggvason 1980; Johnsen et al. 1980; Árnadóttir et al. 1998; Heimisson et al. 2015a).

The spatial distribution of dike openings shows significant first-order regularities (Einarsson 1991; Buck et al. 2006). Several dikes propagated successively mainly in the same direction away from the caldera. Based on their propagation direction, dikes from 1975 to March 1980 can be divided into four sub-sequences (Fig. 1c). Within each sub-sequence, the main dike openings in the fissure swarm manifest a pattern: dikes propagate progressively shorter distances from the Krafla caldera and the length of the main opening also decreases with shorter propagation distance (Fig. 1c). Namely, the first event in each sub-sequence always opens the longest part in the fissure swarm furthest away from the caldera. The pattern is particularly evident for the first sub-sequence events spanning 1975 to January 1977, including the first and largest event of the entire rifting episode. This event most profoundly affected more than 20 km in length of the fissure swarm, > 60 km north of the caldera. The later sub-sequences show the same first-order features with minor deviations (Fig. 1c). After March 1980, the primary character of events shifted to eruptions.

## Methodology

**Fig. 2:**
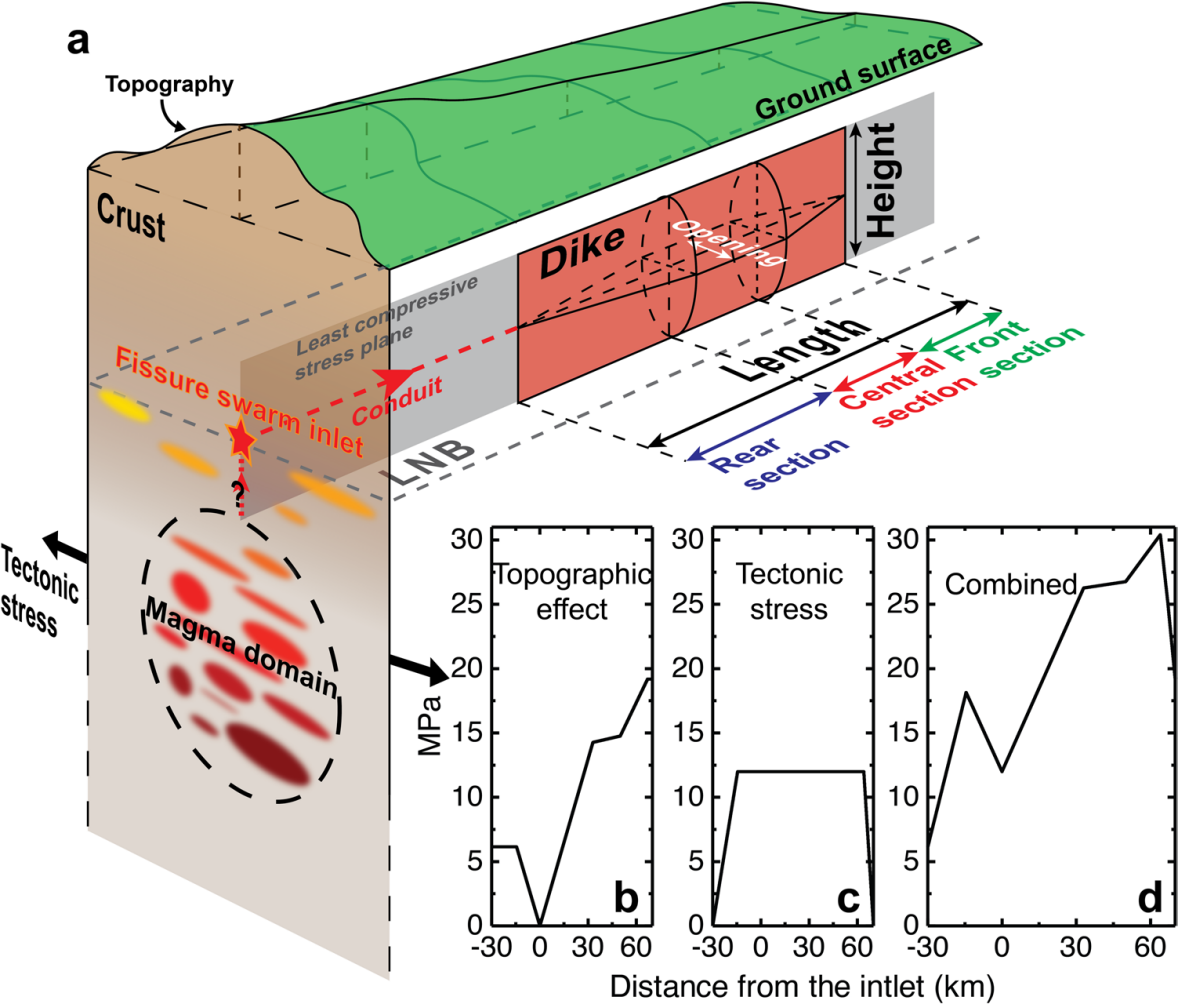
A schematic figure of the revised dike sequence model. **a** Illustration of magma flow from a magma domain, through the fissure swarm inlet and to a dike on the least compressive stress plane. The figure is not to scale. **b**, **c** Dike driving pressure contributed by topographic effects and tectonic stress prior to the 1975–1984 Krafla rifting episode. The topographic effect is calculated with a simplified piecewise elevation profile along the Krafla fissure swarm shown in Fig. 1c and Fig. S3 in Online Resource 1, and an average host rock density of 2510 kg/m^3^ (Scott et al. 2019; see further description in the text). The tensile tectonic stress is assumed to be 12 MPa prior to the first diking event, with two stress barriers on both ends of the Krafla fissure swarm (Buck et al. 2006). **d** Dike driving pressure supplied by the topographic effects and tectonic stress summing up Fig. 2b, c

To reexamine the role of tectonic stress and topography, we develop a revised version of the Buck et al. (2006) model. The model still aims to identify the most favorable part along the fissure swarm for dike opening in each diking event, dependent on tectonic stress, topographic effects, and magma pressure. Our modifications include restricting tectonic stress relieved by each dike and the entire dike sequence, implementing a three-section dike geometry with variable tectonic stress relief, considering of magma compressibility when applying mass conservation as done by Rivalta (2010), and determining the part of dike opening along the fissure swarm according to the stress profile before resolving opening distribution. Using the revised model, we test if the 1975–1984 Krafla dike sequence can be explained by a scenario where magma flow into all the dikes and eruptions originates from approximately the same horizontal location in the fissure swarm, referred to as the fissure swarm inlet (Fig. 2a).

In the model, the fissure swarm inlet is located at the level of neutral buoyancy (LNB) in the crust, where magma density is similar to crustal density (e.g., Rubin and Pollard 1987; Wilson and Head 2002; Poland et al. 2007; Varugu and Amelung 2021). We define magma overpressure as the magma pressure in excess of the lithostatic pressure at the LNB. For simplicity, we define dike opening as its widening perpendicular to the dike plane, dike length as its horizontal scale along the propagation direction, and dike height as its vertical extent. The dike height is assumed to be uniform and centered at the LNB (Fig. 2a). In this study, tensile stress is denoted as negative.

Prior to diking, magma accumulates in a crustal volume we refer to as a magma domain, consisting of partial melt, magma mush and hot solid rock, distributed over a depth interval, as suggested by Sigmundsson et al. (2024). At a certain overpressure, magma begins to flow from the domain and subsequently passes through the inlet to form a dike by lateral flow. Dikes propagate along a plane orthogonal to the direction of least compressive stress (Rubin and Pollard 1988). In the model, tectonic stress accumulation prior to a rifting episode (${\sigma }_{\text{t},\text{total}}$) reduces normal stress on this plane uniformly. Dike intrusions release tectonic stress by causing elastic deformation of the dike flanks (Pollard et al. 1983; Okada 1985). Each diking event thus modifies the stress along the fissure swarm, shifting the location of the most favorable part for the next dike opening. In contrast to the model by Buck et al. (2006), each dike in our model can only release a percentage ($\alpha$) of stored tectonic stress, in line with observations from rifting episodes in Iceland and Afar, east Africa, where repeated lateral dikes occur in the same region (Greiner et al. 2025). This restriction allows the model to predict more realistic dike openings and their associated stress changes.

The model may identify dike openings detached from the inlet under specific stress conditions along the fissure swarm. In such cases, we assume the dike opening is connected to the inlet via a zone with limited opening, referred to as a conduit (Fig. 2a; see also supplementary text in Online Resource 1).

The model is implemented in MATLAB^®^ (The MathWorks Inc. 2023), predicting the key features of a dike sequence, e.g., spatial distribution of dike openings, variations in overpressure at the inlet, and the evolution of tectonic stress along the rift zone. All symbols used in the following derivations are listed in Table 1 and Table S1 in Online Resource 1.

**Table 1:** Parameter settings of the revised dike sequence model for the 1975–1984 Krafla rifting episode. The ‘value’ column indicates the parameter values used to generate the results shown in Figs. 3 and 4. The ‘range’ column specifies the range of values that produces similar spatial distribution of dike openings in the fissure swarm when variating only one parameter and keep others fixed as in the ‘value’ column

Parameter		Value	Range	Reference
Tectonic stress *σ*_*t*, *total*_ [MPa]		−12.0	−11.9 to −12.5	Tryggvason (1984)
Pressure added by upward migration of magma before its lateral propagation (*p*_*u*_) [MPa]		0	/^#^	
Failure limit P_*o*_	First dike [MPa]	15.0	13.9–15.9	
	Rest of dikes* [MPa]	7.5	7.2–7.6	
Host rock (< 5 km depth)	Density *ρ* [kg/m^3^]	2510	2216–2540	Scott et al. (2019)
	Poisson’s ratio *ν*	0.20	0.16–0.22	Heap et al. (2020)
	Shear modulus *μ* [GPa]	10.0	9.4–10.2	Sigmundsson et al. (2024)
Magma domain	Radius *R* [m]	2400	2361–2422	
	Compressibility *β*_*m*_ [Pa^-1^]	2.10 × 10^-10^	1.97 × 10^-10^ to 2.15 × 10^-10^	
Dike	Height *H* [m]	3700	3665–3792	Hollingsworth et al. (2013)
	Central depth [m]	3000	/^#^	
Maximum percentage of tectonic stress relieved by one dike *α*		60%	59%–73%	Tryggvason (1984); Greiner et al. (2025)
Stress reduction ratio between the far end and the local maximum of dike driving pressure *γ*		0.30	0.13–0.30	
Tapering factor *ε* [Pa/m]		130	80–136	

^#^*p*_*u*_ only changes the inlet overpressure at dike initiations but does not affect the spatial distribution of predicted dike openings. Central depth of dikes only determines the absolute pressure of dike and the inlet but does not affect the spatial distribution of predicted dike openings

*The ratio between the failure limits for the first and rest of dikes is constrained by the elevation curve observed during the Krafla rifting episode, e.g., Einarsson (1991)

To rupture the magma domain and initiate magma flow, magma overpressure in the domain should increase sufficiently that it equals the sum of the host rock failure limit (${P}_{\text{o}}$; e.g., tensile strength) and the external stress (e.g., Blake 1981; Zhan and Gregg 2019; Sigmundsson et al. 2020). Overpressure in the domain results from magma supply into a confined volume, as well as from the buoyancy generated by density contrast between magma and host rock. Assuming that tectonic stress is the only contributor to external stress and that it is uniform with depth, the magma overpressure at the inlet (${p}_{\text{i}}$) at dike initiations is expressed as (Buck et al. 2006; Qin and Buck 2008):

1 $${p}_{\text{i}}={P}_{\text{o}}+{\sigma }_{\text{t},\text{i}}+{p}_{\text{u}}$$

where ${\sigma }_{\text{t},\text{i}}$ represents the tectonic stress at the inlet (negative for tension), and ${p}_{\text{u}}$ accounts for any potential overpressure added by magma upward migration before its lateral propagation (Sigmundsson et al. 2020). Assuming the upward migration is the same during all dike initiations in a rifting episode, ${p}_{\text{u}}$ is treated as constant. The failure limit is adjustable and is set higher for the first dike than for later dikes, in line with elevation change curves from rifting episodes (Tryggvason 1980; Parks et al. 2025).

After a lateral dike is initiated, the driving pressure for magma flow is contributed by both inlet overpressure and the stress conditions along its path on the least compressive stress plane. The initial magma overpressure is controlled by Eq. (1). The stress conditions along the dike path are determined by two factors: (i) topographic overburden from difference in lithostatic stress between the inlet and the dike path (e.g., Fialko and Rubin 1998; Sigmundsson et al. 2015; Urbani et al. 2017). Using the peak elevation along the Krafla fissure swarm as a reference (latitude of ~ 65.734° N in Fig. 1c), all other points along the profile have negative relative stress due to smaller lithostatic stress, increasing the driving pressure of each dike (Fig. 2b); (ii) accumulated tensile tectonic stress along the dike path that reduces stress acting perpendicularly to the propagating dike (Fig. 2c). The net driving pressure of lateral dike propagation, ${P}_{\text{driving}}$, is given by

2 $${P}_{\text{driving}}={p}_{\text{i}}-\rho g\Delta h(x)-{\sigma }_{\text{t}}(x),$$

where $x$ indicates any location in the dike, $\rho$ is average host rock density above the central depth of dikes, $\Delta h(x)$ is the elevation difference above the inlet and other locations along the fissure swarm, ${\sigma }_{\text{t}}(x)$ is the tectonic stress at each horizontal location along the dike path, and $g$ is the gravitational acceleration. Considering the minimum potential energy principle and that dikes take the path of least resistance (Heimisson et al. 2015b), dikes in the model will propagate in the direction with larger positive gradient of ${P}_{\text{driving}}$ near the inlet, continuing towards an area where ${P}_{\text{driving}}$ reaches its local maximum and no longer increases. With magma extracted from the magma domain, ${P}_{\text{driving}}$ decreases due to reduction in source overpressure (${p}_{\text{i}}$ decreases) and in tectonic stress (${\sigma }_{\text{t}}$ becomes less negative). Dike intrusion stops when ${P}_{\text{driving}}$ drops to zero, achieving equilibrium between overpressure at the inlet and the dike.

### Stress change scaling with dike openings

Our model assumes that dikes release accumulated tectonic stresses elastically, such that the opening at each mathematically discretized location of the dike scales with the stress release, in accordance with Hooke’s law. We derive a scaling relationship (see derivation in Online Resource 1) between average dike opening at each horizontal location, $w(x)$, and change in tectonic stress at that location, ${\Delta \sigma }_{\text{t}}(x)$, such that

3 $$w(x)=A\frac{\pi H\left(1-\nu \right)}{4\mu }{\Delta \sigma }_{\text{t}}(x),$$

where $\nu$ and $\mu$ represent the Poisson’s ratio and shear modulus of the host rock, respectively, $H$ indicates the dike height, and *A* is a constant. The term $\frac{\pi H\left(1-\nu \right)}{4\mu }$ is the scaling factor between average opening and stress change of a two-dimensional elliptical crack in an unbounded elastic material (Pollard et al. 1983). However, dikes forming in a rifting episode are highly influenced by their location close to the free surface of the Earth, causing larger opening than anticipated in the full space approximation. Dike opening models (Online Resource 1) suggest $A\sim 2$, used in this study.

Equation (3) with $A=2$ can be used to derive a relation between the mass (or volume) of a dike and the stress release, assuming negligible variation of magma density within the dike:

4 $${\Delta M}_{\text{d}}={\rho }_{\text{d}}{\int }_{\text{L}}Hw(x){\text{d}}x\approx {\rho }_{\text{d}}\frac{\pi \Delta L{H}^{2}\left(1-\nu \right)}{2\mu }{\sum }_{\text{L}}{\Delta \sigma }_{\text{t}}(x)$$

Here $L$ is the dike length, and ${\rho }_{\text{d}}$ is the magma density in the dike. Integrating the horizontal cross-section area $Hw(x)$ over the dike gives the dike volume, corresponding to the discretization as presented above.

### Coupling of dikes and magma domain with mass conservation and elasticity

We infer the variation of ${P}_{\text{driving}}$ by considering the coupling between a dike and a magma domain through the inlet, mass conservation, and elasticity. Mass conservation implies that the magma mass extracted from the magma domain (${\Delta M}_{\text{s}}$, the source) equals to that injected into a dike (${\Delta M}_{\text{d}}$):

5 $${-\Delta M}_{\text{s}}={\Delta M}_{\text{d}},$$

where ${\Delta M}_{\text{s}}$ is negative and ${\Delta M}_{\text{d}}$ is positive during dike emplacement.

Assuming no additional feeding into the magma domain during diking, loss of mass in the magma domain leads to its pressure decrease, reducing both its volume and density as the remaining material within it expands. We assume the inlet overpressure drop (${\Delta p}_{\text{i}}$) is equivalent to the overpressure drop in the magma domain. Assuming the variations of temperature and composition are second order effects, then

6 $${\Delta M}_{\text{s}}={\rho }_{\text{s}}{V}_{\text{s}}\left({\beta }_{\text{m}}+{\beta }_{\text{s}}\right){\Delta p}_{\text{i}},$$

where ${\rho }_{\text{s}}$ and ${V}_{\text{s}}$ are the average density of material within the magma domain and the total volume of the magma domain, respectively (e.g., Segall et al. 2001; Rivalta 2010; Sigmundsson 2021). The effective compressibility of material within the magma domain, ${\beta }_{\text{m}}$, and the so-called mechanical compressibility of the magma domain structure within elastic host rock, ${\beta }_{\text{s}}$, together relate the mass change in the magma domain with its volume and pressure variations, where ${\beta }_{\text{m}}$ ranges 0.04–1.5 GPa^−1^ (Spera 2000; Segall et al. 2020; Sigmundsson et al. 2020). Assuming the host rock is a homogeneous elastic half-space and approximating the magma domain as a sphere with radius $R$ (volume ${V}_{\text{s}}=4\pi {R}^{3}/3$), then ${\beta }_{\text{s}}=3/4\mu$ (McTigue 1987).

Equations (4), (5), and (6) can be combined to couple together the variations of magma overpressure at the inlet and tectonic stress change in the fissure swarm:

7 $${\Delta p}_{\text{i}}=-\frac{{\rho }_{\text{d}}}{{\rho }_{\text{s}}}\frac{{H}^{2}\Delta L\left(1-\nu \right)}{2{R}^{3}\left(1+\frac{{\beta }_{\text{m}}}{{\beta }_{\text{s}}}\right)}{\sum }_{\text{L}}{\Delta \sigma }_{\text{t}}(x)$$

The volume ratio between the dike and the deflation of a magma domain is $1+\frac{{\beta }_{\text{m}}}{{\beta }_{\text{s}}}$ (Rivalta 2010). For any location $x$ in a dike where the driving pressure decreases to zero at the end of the intrusion, the equilibrium condition can be written as

8 $${P}_{\text{driving}}^{0}-{\Delta \sigma }_{\text{t}}(x)+{\Delta p}_{\text{s}}=\left({p}_{\text{i}}+{\Delta p}_{\text{i}}\right)-\rho g\Delta h(x)-{\sigma }_{\text{t}}^{0}(x)-{\Delta \sigma }_{\text{t}}(x)=0$$

where ${P}_{\text{driving}}^{0}$ represents dike driving pressure at its initiation and ${\sigma }_{\text{t}}^{0}(x)$ is the initial tectonic stress along the dike path prior to the diking event. The terms ${p}_{\text{i}}+{\Delta p}_{\text{i}}$ give the final inlet overpressure.

### Three-section dike geometry and numerical implementation of the model

Implementing the above equations requires specifying the dike geometry. We use a simplified geometrical form, with more opening in a central section and less near dike ends, similar as dike opening under constant overpressure in an unbounded elastic material (Pollard et al. 1983). The model divides a lateral dike into three sections: front, central, and rear (Fig. 2a and Fig. S2 in Online Resource 1). The dike propagates along a positive gradient of ${P}_{\text{driving}}$ to the location where ${P}_{\text{driving}}$ reaches its local maximum. There, (i) the dike has its maximum opening, and (ii) the largest reduction in tectonic stress occurs. Beyond this point, dike opening tapers toward its far end because the driving pressure continually drops towards the front. This effect is implemented by prescribing a linear decrease in tectonic stress release, with a tapering factor ($\varepsilon$) being the slope. Dikes will stop propagating when the driving pressure drops below a certain level (Buck et al. 2006), implemented here as the tectonic stress release reduced to a preset fraction ($\gamma$) of ${\Delta \sigma }_{\text{t},\text{max}}$, or to zero in cases of a rapid pressure drop in the front section induced by topographic gradients or stress barriers.

The rear section begins at the dike’s near end and extends away from the inlet (Fig. 2a). Tectonic stress reduction within this section is variable such that the final dike driving pressure from external contributors becomes uniform, determined by solving for ${\Delta \sigma }_{\text{t}}(x)$ in Eq. (8). In this model, any individual dike cannot reduce tectonic stress by more than a fixed maximum percentage of the total tectonic stress stored prior to the rifting episode ($\alpha {\sigma }_{\text{t},\text{total}}$). Namely, at any location where ${\Delta \sigma }_{\text{t}}\left(x\right)>\alpha {\sigma }_{\text{t},\text{total}}$, we set ${\Delta \sigma }_{\text{t}}(x)=\alpha {\sigma }_{\text{t},\text{total}}$. This yields a section of uniform opening, referred to as the central section. It is typically located further away from the inlet than the rear section. The final geometry of the dike is determined by searching for ${\Delta \sigma }_{\text{t}}(x)$ in the rear section so that equilibrium in Eq. (8) is achieved. That specifies the dike opening, tectonic stress reduction, and the lengths of the three sections (details in Online Resource 1).

For numerical implementation, we discretize the horizontal axis of the dike propagation plane with interval $\Delta L$. When the critical limit in Eq. (1) is reached, a numerical algorithm evaluates the local maxima of ${P}_{\text{driving}}$ and searches for ${\Delta \sigma }_{\text{t}}(x)$ in the dike rear section. The hydraulic equilibrium between the inlet and the rear section can be written according to Eqs. (7) and (8) for any location $x$ in the rear section:

9 $${p}_{\text{i}}-\frac{{\rho }_{\text{d}}}{{\rho }_{\text{s}}}\frac{{H}^{2}\Delta L\left(1-\nu \right)}{2{R}^{3}\left(1+\frac{{\beta }_{\text{m}}}{{\beta }_{\text{s}}}\right)}{\sum }_{\text{L}}{\Delta \sigma }_{\text{t}}\left(x\right)=\rho g\Delta h(x)+{\sigma }_{\text{t}}^{0}(x)+{\Delta \sigma }_{\text{t}}(x)$$

The model inputs include the profiles of topography and tectonic stress along the dike path, along with parameters listed in Table 1. The model tests values of ${\Delta \sigma }_{\text{t}}(x)$ in the rear section and their corresponding dike geometry aiming at fulfilling the condition described in Eq. (9), and from that, derives the distribution of dike opening. The tectonic stress profile is updated considering the stress released by one dike and used as input for the next dike. The modeling steps are (demonstrated by Fig. S2 in Online Resource 1 and the animation in Online Resource 2):

(i) Calculate initial magma overpressure with Eq. (1) and locate where ${P}_{\text{driving}}$ reaches its local maximum nearest to the fissure swarm inlet;
(ii) Set ${\Delta \sigma }_{\text{t}}(x)=-{\alpha \sigma }_{\text{t},\text{total}}$ at the location found in (i);
(iii) Determine dike geometry with ${\Delta \sigma }_{\text{t}}(x)$ and evaluate Eq. (9);
(iv) If the far end locates in an area with positive gradient of driving pressure, move to the next local maximum of ${P}_{\text{driving}}$ further away from the inlet and go back to (ii);
(v) If the left-hand side in Eq. (9) is larger, increase ${\Delta \sigma }_{\text{t}}(x)$ by a certain searching step (0.05 MPa in this study) and go back to (iii);
(vi) If the left-hand side in Eq. (9) is smaller, decrease ${\Delta \sigma }_{\text{t}}(x)$ by the searching step and go back to (iii);
(vii) If equilibrium in Eq. (9) is fulfilled, stop the search, and all information of the dike is now available;
(viii) Update the tectonic stress along the fissure swarm and go back to (i) for the next dike intrusion.

One can decide which parameters in Table 1 to fix, depending on available a priori information. In this study, the sensitivity of the free parameters is tested by changing one parameter at a time and evaluating how the spatial distribution of dike openings is modified (see parameter sensitivity analysis in discussion).

### Eruption

Equation 1 suggests that higher inlet overpressure is required to initiate dikes in the later stage of a rifting episode compared to the early stage, as tectonic stress near the fissure swarm inlet is progressively reduced by dike intrusions. This conforms to the ground uplift at dike initiations after the first dike intrusion during the Krafla rifting episode.

Increased initial magma overpressure at the inlet may drive upward magma movement and cause eruptions. In our model, eruption occurs when inlet pressure is high enough to drive magma flow, yet significant tectonic stress relief from previous dike intrusions reduces the support for lateral propagation. In step (iii) of the numerical algorithm, a simple condition for eruptions is added:

10 $${-\Delta p}_{\text{i}}\le \left(1-\gamma \right){p}_{\text{i}}{\text{ and }}{\sigma }_{\text{t},\text{i}}<\left(1-\gamma \right){P}_{\text{o}}$$

where ${\Delta p}_{\text{i}}$ is evaluated with the dike geometry in step (iii). When the overpressure at the inlet drops to $\gamma {p}_{\text{i}}$, the eruption will stop. Dikes that feed eruptions are assumed to only have a front section tapering from the inlet, thus limiting its propagating distance. Due to effects of faulting, as well as variations in physical properties of magma and host rock above the assumed dike top (e.g., Segall et al. 2001; Huppert and Woods 2002; Taisne and Jaupart 2009; Hollingsworth et al. 2013; Magee and Jackson 2020; Townsend and Hubber 2020), our model cannot predict the location of eruptive fissures. Furthermore, predicted dike openings with eruptions are less accurate than those without eruptions.

## Results

**Fig. 3:**
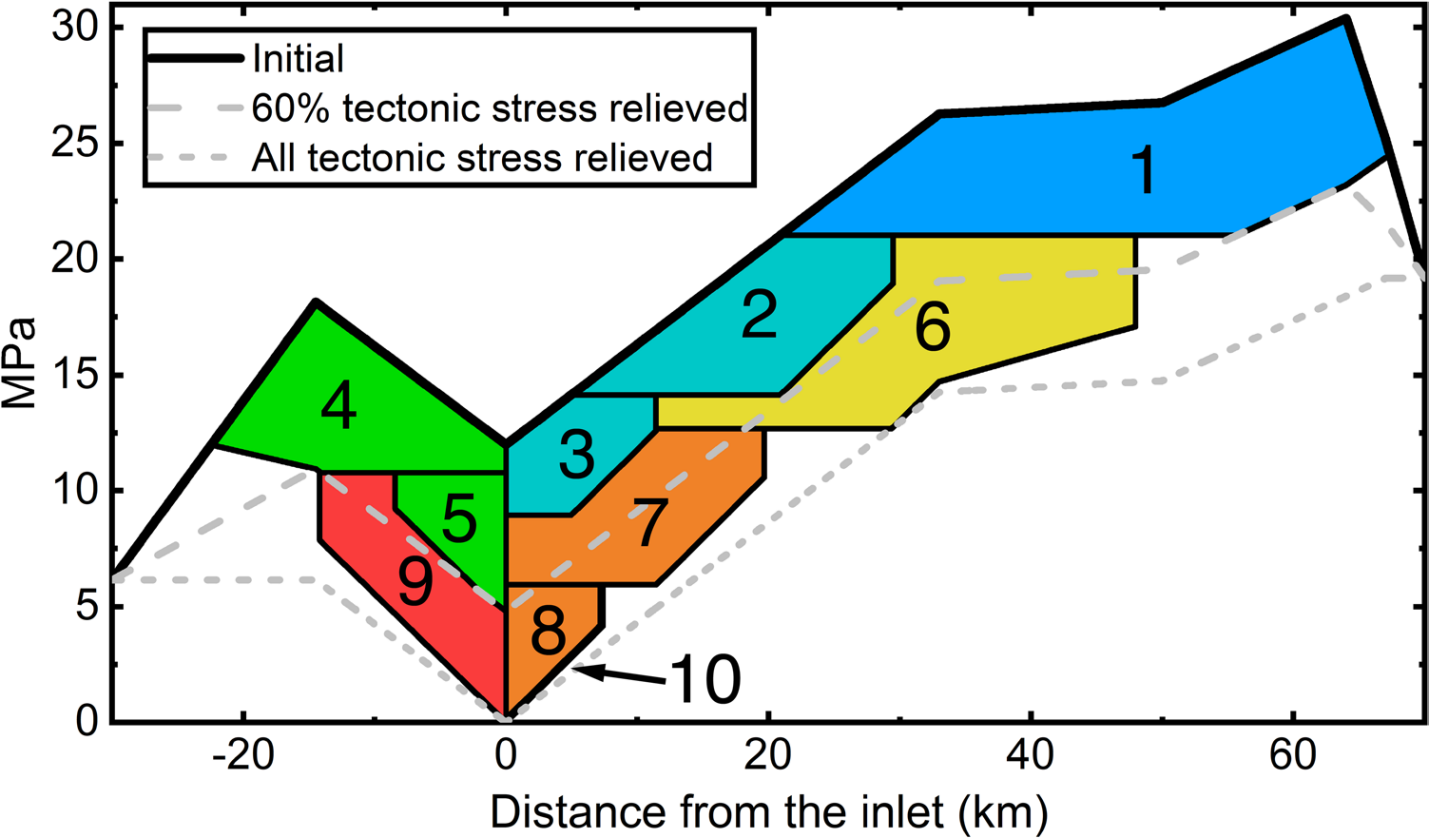
Variation in dike driving pressure contributed by topographic effects and tectonic stress during the 1975–1984 Krafla rifting episode, predicted by the revised dike sequence model. The numbers indicate the temporal order of the dikes. The thick black line corresponds to the curve in Fig. 2d. The color codes correspond to Fig. 4d. An animation in Online Resource 2 demonstrates the spatio-temporal sequence of diking which contributes to the cumulative data displayed in the figure

**Fig. 4:**
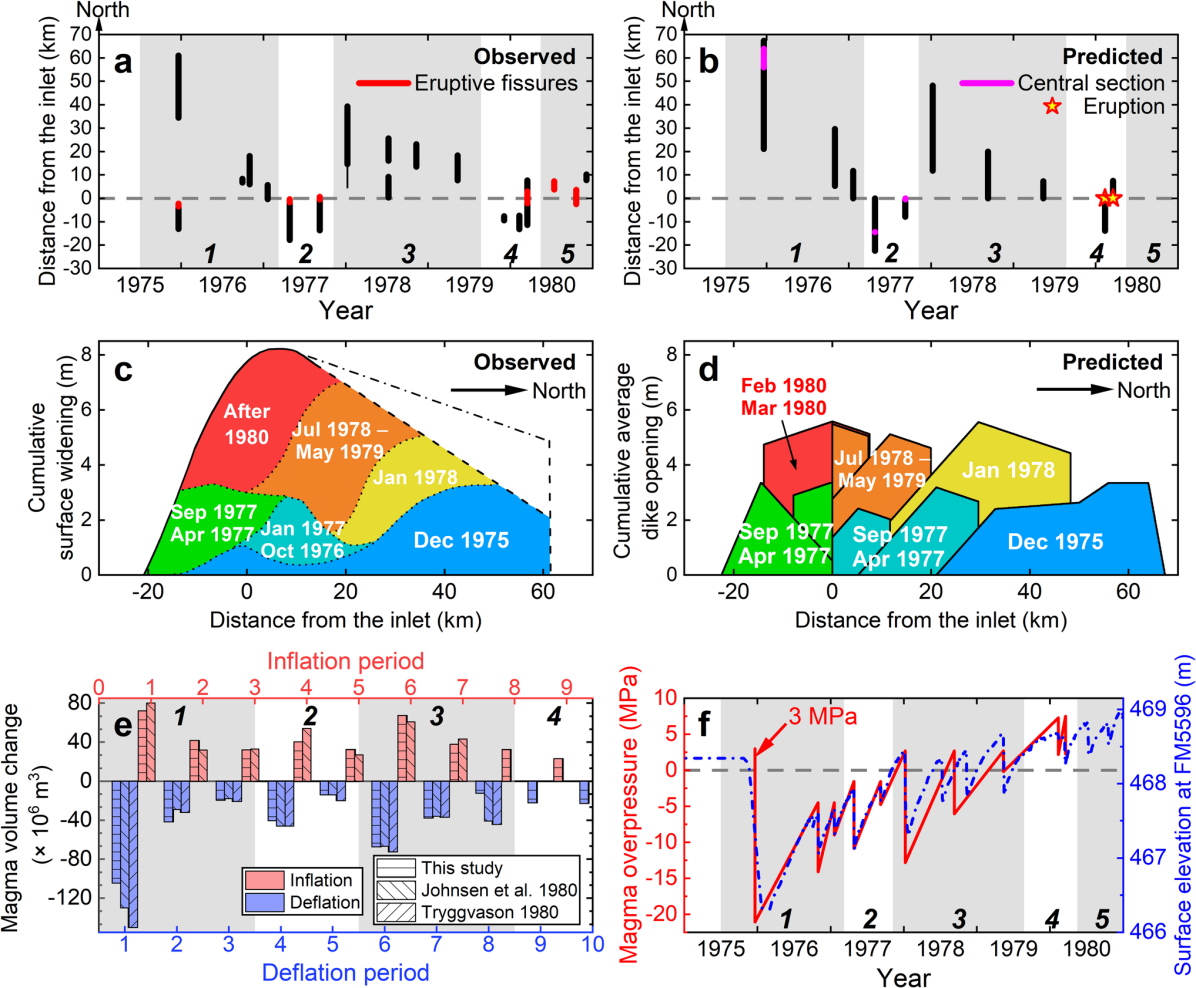
Comparison between predictions from the revised dike sequence model and geophysical observations and previous models of the 1975–1984 Krafla rifting episode. **a** The distance range in the Krafla fissure swarm with main dike opening (black) indicated by seismic swarms and eruptions (red), adapted from Einarsson (1991) and Buck et al. (2006), same as Fig. 1c. The vertical white and grey straps represent different sub-sequences of dike intrusions numbered at the bottom. The inlet is located 4 km north of the center of the caldera. **b** Spatial distribution of dike openings predicted by the revised dike sequence model. **c** Estimated cumulative surface widening in the fissure swarm with distance measurements, adapted from Tryggvason (1984). **d** Predicted cumulative dike opening (average over the depth range of diking) in the fissure swarm. The color codes correspond to estimates for different period in Fig. 4c. **e** Volume change in the magma domain, predicted by the revised dike sequence model and inverted with a point pressure source within uniform elastic half-space (Mogi 1958) using ground deformation (Johnsen et al. 1980; Tryggvason 1980). **f** Magma overpressure at the inlet (red line) predicted by the revised dike sequence model and observed elevation change in the Krafla caldera (blue dash-dot line; Tryggvason 1984). The horizontal grey dashed line indicates magma pressure equals to lithostatic pressure. Overpressures after each event and at the initiation of next event is connected by straight lines without any indication of dynamic processes of dike emplacement or recharge of the magma domain

We evaluate if the first ten dikes of the 1975–1984 Krafla rifting episode, covering four sub-sequences with most surface widening in the fissure swarm, can be explained by the revised dike sequence model. The fissure swarm inlet is assumed to be below the highest elevation along the Krafla fissure swarm in the caldera. The tensile tectonic stress is assumed to be 12 MPa with two stress barriers on both ends of the Krafla fissure swarm (Buck et al. 2006; Fig. 2c). Table 1 lists parameter values we use, constrained by previous research where available and otherwise tested over a range of values to reproduce the observed dike opening distribution (Fig. 1c). The density ratio ${\rho }_{\text{d}}/{\rho }_{\text{s}}$ is set to 1 in Eq. (9), assuming the difference between the magma density in the magma domain and dikes is negligible. The failure limit ${P}_{\text{o}}$ in Eq. (1) to initiate the first dike is set larger than ${P}_{\text{o}}$ for later dikes in the Krafla rifting episode, scaled according to the ground elevation in the Krafla caldera (Fig. 1d; Einarsson 1991). The maximum percentage of stored tectonic stress that a single dike can release is inferred from surface widening estimates by Tryggvason et al. (1984). Results in Figs. 3 and 4 are from model runs using parameter values in Table 1.

Figure 3 shows the external contributors to dike driving pressure at the onset of the rifting episode and how it evolves with dike intrusions. External factors contribute up to 30 MPa to the driving pressure for the first dike and ~20 MPa for the second and sixth dikes. Dikes relieve tectonic stress and reduce the driving pressure available for later events (see the animation in Online Resource 2). Assuming initial magma overpressure at the inlet is 3 MPa prior to the rifting episode, the model suggests that the topographic effect and tectonic stress account for >90% of the dike driving pressure for its lateral propagation in the early stage of the rifting episode. Tectonic stress relieved by preceding dikes limits the propagation distance of later dikes by shifting the local maxima of dike driving pressure.

Other predictions by the revised dike sequence model are compared with geophysical observations and previous models in Fig. 4. The predicted dike opening distribution and inlet overpressure changes are compared to the Buck et al. (2006) model in Fig. S4 in Online Resources 1. Generally, the predicted dike opening distribution aligns with that indicated from observations (Fig. 4a, b). Dike intrusions from 1975 to 1977 generated ~ 3 m of surface widening over ~ 80 km along the fissure swarm, and dikes from 1978 to 1979 caused again ~ 2–3 m of surface widening north of the fissure swarm inlet (Fig. 4c, d). The largest cumulative surface widening near the inlet is ~ 6 m in Fig. 4d, smaller than ~ 8 m inferred from ground observations, as shown in Fig. 4c. The model predicts eruptions associated with dike intrusions after 1979 (Fig. 4b) when tectonic stress near the inlet has been mostly relieved (Fig. 3). Figure 4e suggests that the revised dike sequence model, even without direct constraints from ground deformation data, predicts magma volume change in the magma domain that is consistent with geodetic models. Variations of surface elevation and magma overpressure at the inlet in Fig. 4f show similar inflation–deflation cycles and a general increase at dike initiations, except for the first one, as observed during the rifting episode.

## Discussion

### Implications for the Krafla rifting episode

Comparisons above indicate that the model successfully reproduces the first-order regularities of the 1975–1984 Krafla dike sequence. A relatively low magma overpressure of 3 MPa at the fissure swarm inlet prior to the rifting episode can generate the first and largest dike intrusion of the entire sequence, dominated by topographic effects and tectonic stress (Fig. 3). By 1979, the tectonic stress near the inlet had been mostly relieved, and dikes thereafter are likely to cause eruptions. The dikes after March 1980 are not modeled due to less accurate opening prediction with the involvement of eruptions (see “Eruption” section). Comparing the model-predicted dike openings with the surface widening estimated with the geodetic approach shows that, after 1980, about 25% of cumulative widening occurred in the zone near the magma inlet associated with the unmodeled dikes.

Figure 4f shows that a higher failure limit is required to initiate the first dike than later dikes by a factor of 2, which fits the observed elevation change. Since the first dike intrusion did not significantly reduce the tectonic stress near the inlet, a lower failure limit for later dikes permits their initiations under negative magma overpressure at the inlet, namely when magma pressure is lower than the lithostatic pressure. The predicted inlet overpressure variations (red curve in Fig. 4f) align with the observed elevation changes (blue curve in Fig. 4f). Based onthe predicted inlet overpressure variations, we find that a spherical pressure source with a radius of 2.4 km (Table 1) in an elastic half-space (Mogi 1958) at ~ 3.6 km depth leads to a similar magnitude of elevation changes as the observations. This is consistent with previous geodetic inversions (e.g., Tryggvason 1980; Johnsen et al. 1980; Ewart et al. 1991; Heimisson et al. 2015a).

### The magma domain and the location of the fissure swarm inlet

For the Krafla rifting episode, our model suggests that the fissure swarm inlet is offset from the magma domain center, similarly as found for the ongoing Svartsengi rifting episode in Iceland (Sigmundsson et al. 2024). Both our model and the Buck et al. (2006) model (Fig. 4a, b and S4a in Online Resource 1) indicate that an inlet located under the highest surface elevation along the fissure swarm, ~ 3 km north of the Krafla caldera center, explains the spatial distribution of dike openings. This area coincides with the center of the 1975–1984 eruptive fissures (Fig. 1c), the epicenters of seismicity in the beginning of intrusive activities during 1978–1980 (~ 67.5° N; Einarsson and Brandsdóttir 2021), and the boundary of bimodal eruptive products across the north caldera rim, where magma mixing and supply from different depths occurred (Rooyakkers et al. 2024). In contrast, geodetic models suggested that the magma domain center is near the caldera center, e.g., with deformation source centers located within 1 km of it (Heimisson et al. 2015a). The offset between the fissure swarm inlet and the magma domain center may result from structural weakness along caldera-bounding faults and local stress regime below caldera rims (Sigmundsson et al. 2020; Corbi et al. 2016).

For the model of Krafla rifting episode presented here, the upward migration of magma before its lateral propagation is assumed to be negligible (${p}_{\text{u}}$ being zero), as the point-source pressure at ~ 3–4 km depth, inferred from geodetic models and Fig. 4f, is close to the assumed LNB at central depth of the dikes (3 km in Table 1). We also vary the value of ${p}_{\text{u}}$ in the sensitivity test, which does not alter the predicted dike opening patterns but only shifts the estimate of overpressure as a whole.

Magma overpressure change at the fissure swarm inlet is assumed to be the same as the pressure change in the magma domain, represented in our model by a single spherical volume with a radius of 2.4 km. Petrochemistry analyses of eruptive products during the Krafla rifting episode (Rooyakkers et al. 2024) have, however, inferred basaltic magma storage prior to the Krafla eruptions at both ~ 7–9 km (shallow) and ~ 14–19 km (deep). Dike intrusions tapped the shallow and deep storage in a different manner during individual eruptions of Krafla, and primitive magma erupted north of the caldera flowed from the deep storage zone directly to the surface without being stored in the shallow zone. We suggest this distributed storage of basaltic magma may correspond to a magma domain ranging over a depth interval of ~ 5–12 km. In this case, a prolate ellipsoidal volume, with a larger vertical extent than a sphere with a radius of 2.4 km, may better represent the magma domain. Anderson and Segall (2011) demonstrated that the mechanical compressibility difference between a sphere and a prolate ellipsoid within an elastic half-space is less than 25%, whereas the difference between a sphere and a penny-shaped crack is two orders of magnitude. Assuming a prolate ellipsoidal magma domain rather than a spherical one is unlikely to affect the first-order regularities of the dike sequence behavior and variations in inlet overpressure.

### Parameter sensitivity analysis

Equation 9 indicates that most parameters in the model are correlated, among which the most simple and straightforward correlation is between the radius and compressibility of the magma domain. Changing one parameter value may be accommodated by adjusting one or more other parameters to obtain a similar spatial distribution of dike openings. Thus, it is important to constrain as many parameters as possible from previous research (Table 1). The failure limit explored in this study is in the range of ~ 1–20 MPa, comparable to tensile strength indicated by laboratory rock tests (Zhan and Gregg 2019) and dike tensile strength of < 10 MPa constrained by geodetic observations for large-scale strength of rocks inferred by Jónsson (2012). Poisson’s ratio in Table 1 is close to the average value in Heap et al. (2020), and its range in Table 1 overlaps largely with values from previous models or experimental data. Shear modulus of 10 GPa has been applied for other volcanological models in Iceland (e.g., Hartley and Maclennan 2018; Juncu et al. 2020; Sigmundsson et al. 2024). Dike height is constrained with ground deformation by Hollingsworth et al. (2013).

We evaluate in particular the sensitivity of free parameters to validate the conclusion that a relatively low magma overpressure at a fissure swarm inlet prior to the Krafla rifting episode can broadly explain the dike sequence behavior. We adjust one individual parameter at a time and fix other parameters at their plausible values in Table 1. The parameter ranges in Table 1 are obtained by testing when the dike opening distribution (Fig. 4b) changes significantly, e.g., any dike propagates in the other direction, or the dikes propagate out of the range of the fissure swarm.

The parameter ranges indicate the model is least sensitive to dike tapering factor $\varepsilon$ and pressure change ratio $\gamma$ since the volume of the front section is small compared to the dike length. The host rock density, which controls the magnitude of dike driving pressure from the topographic effects, also has little influence on the dike pattern prediction. The density range in Table 1 is within a larger range of rock density ~ 1800–2700 kg/m^3^ for the Krafla area inferred by petrological and geophysical studies (Eggertsson et al. 2020; Darbyshire et al. 2000). Different assumptions of rock density do not alter the significance of topographic effects driving long-distance dike propagation, and similar dike opening distributions are obtained. Our parameter sensitivity analysis shows that the maximum percentage of tectonic stress relieved by an individual dike ($\alpha$) should be less than 73%, indicating that individual dikes release only part of the tectonic stress accumulated prior to a rifting episode. Nevertheless, varying the value of $\alpha$ between 59% and 73% does not change the model predictions significantly, since it is not the only factor limiting dike openings, e.g., the size of the magma domain also contributes (Buck et al. 2006).

Our model demonstrates that initially low magma overpressure at the inlet may broadly explain the dike sequence behavior during the 1975–1984 Krafla rifting episode, where the failure limit and tectonic stress play important roles. The failure limit determines the initial disequilibrium in the inlet-dike system in Eq. (9). A higher failure limit requires a larger volume of magma extracted from the inlet to resume equilibrium, influencing the dike opening distribution. With the same percentage of tectonic stress relieved, higher accumulated tectonic stress prior to the rifting episode leads to more lateral dikes and predicts no eruption for the first ten events in the Krafla rifting episode. In Table 1, the highest value of the failure limit of 15.9 MPa for the first dike and the lowest value of tensile tectonic stress of 11.9 MPa still give an inlet overpressure of only 4 MPa prior to the rifting episode. Further trials of parameter combinations give a maximum possible magma overpressure of ~ 10 MPa prior to the rifting episode. The ratio of 1.8–2.2 between the failure limits of the first and later dikes leads to a similar prediction of dike opening distribution. This parameter controls the inlet overpressure difference at the initiations of the first and second dikes, where a comparison between observed and predicted elevation change in Fig. 4f favors a ratio of 2.

### The case of the 2023–2025 Svartsengi rifting episode

**Fig. 5:**
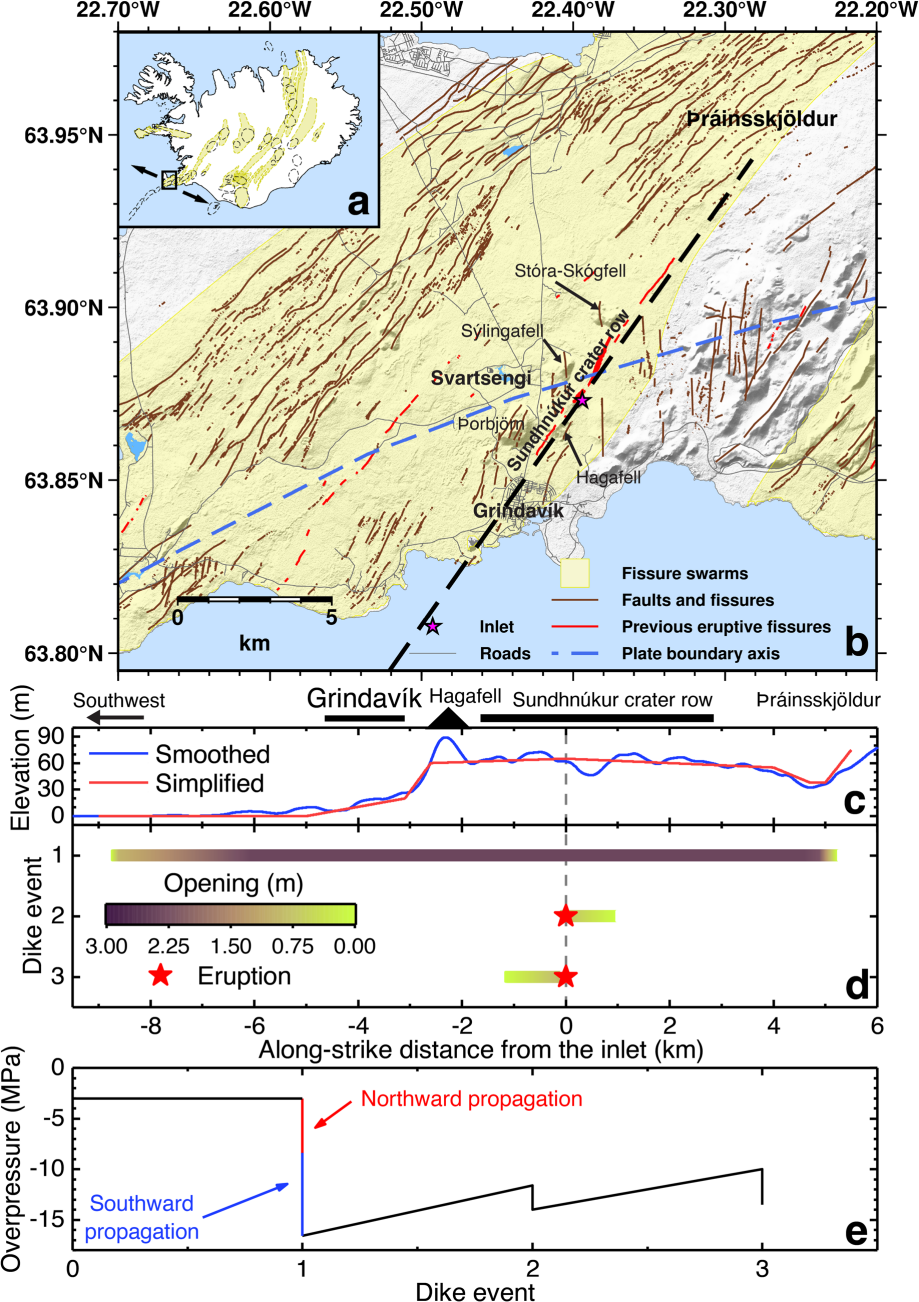
A plausible dike sequence model for the first three dikes of the 2023–2025 Svartsengi rifting episode, SW Iceland. **a** A map of Iceland illustrating the location of the Svartsengi area with the black rectangle. The arrows indicate the direction of plate spreading in the Svartsengi area. **b** A map of the Svartsengi area. The black dashed line indicates the approximate strike of the dike intruded in November 2023 from Sigmundsson et al. (2024). Þráinsskjöldur is a lava shield formed around 14,100 years ago with general higher elevation than its surroundings (Jónsson 1978). The faults and fissures are from Clifton and Kattenhorn (2006), the fissure swarms are from Jóhannesson and Sæmundsson (2009), the plate boundary axis is from Sigmundsson et al. (2024) and the topography is from LMÍ (2020). **c** Elevation profile along the strike of the first dike. The smoothed one is derived from the real topography with Savitzkyp-Golay filter. **d** The distribution of dike openings predicted by the revised dike sequence model for the three dike intrusions. **d** Variation of overpressure from the magma supply into the magma domain predicted by the revised dike sequence model

A dike sequence similar to the 1975–1984 Krafla rifting episode has occurred at the Sundhnúkur crater row in the Svartsengi volcanic system, SW Iceland (Fig. 5; Sigmundsson et al. 2024; Parks et al. 2025). The first dike intrusion formed a ~ 14-km-long dike in November 2023, ~ 6 km to the north and ~ 8 km from a fissure swarm inlet inferred from all seven dike intrusions during November 2023 and July 2024 (Fig. 5b; Parks et al. 2025). Eight more dike intrusions with seven eruptions manifest alternations between southward and northward propagation.

We applied our revised dike sequence model to the first three events of the rifting episode. Our model inherently assumes unidirectional propagation. However, for the first dike at the Sundhnúkur crater row, the ratio between the northeast and southwest propagation distances is 0.75 (6 km over 8 km), compared to ~ 0.25 between the southward and northward propagation of the first dike at Krafla (~ 15 km over ~ 60 km). Hence, the first dike is modeled with two separate iterations in the numerical implementation—once the central section of the dike reaches the fissure swarm inlet, all remaining overpressure at the inlet is used to calculate the dike opening in the other direction. This is only to predict the final opening distribution of such bilateral dike propagation without any indication of its temporal evolution. The initial propagating direction is imposed by setting the elevation gradient slightly steeper to the north since later events of the rifting episode propagate more to the north (Parks et al. 2025). Topographic stresses are calculated from a simplified elevation profile along the strike of the first dike (Fig. 5c). The parameter settings of one plausible model are listed in Table S2 in Online Resource 1.

Figure 5d, e shows the predictions of the model. Tectonic stress reductions with dike intrusions are presented in Fig. S5 in Online Resource 1. The part in the fissure swarm opened by the first dike is predicted to be 14 km long with the average opening up to ~ 3 m (Fig. 5d), in the magnitude range of ~ 2–7 m by geodetic inversions (Sigmundsson et al. 2024). The second and third events propagate to the north and south, respectively, consistent with the locations of eruptive fissures (Parks et al. 2025). The predicted overpressure variation at the inlet in Fig. 5e indicates that the northward propagation of ~ 5 km reduces ~ 5.3 MPa of inlet overpressure, while the southward propagation of ~ 9 km reduces ~ 8.2 MPa. Considering simple proportion to the propagation distances, the pressure drop from the southward propagation is expected to be ~ 9.5 MPa. A relatively low pressure drop from the southward propagation predicted by the model may relate to the elevation decrease northeast of Grindavík, which contributes some 2 MPa of dike driving pressure, highlighting the importance of topographic effects. In this model, a maximum buoyancy effect of 9 MPa (Sigmundsson et al. 2024) and tectonic stress allow dike initiations when overpressure from magma supply into the domain is ~ −3 MPa. If the accumulated tectonic stress prior to the rifting episode is smaller than 7.7 MPa, the model predicts eruption already for the first dike intrusion. These again highlight the contributions of topography to dike propagation distances, the importance of tectonic stress for relatively low magma overpressure at the inlet to initiate dikes, and possibly for the absence of eruptions in the initial event.

Overpressure added by magma upward migration before its lateral propagation (${p}_{\text{u}}$) is 6 MPa in this case compared to zero in the model for the Krafla rifting episode as presented above. Geodetic models inferred that the magma domain at Svartsengi is ~ 2 km below the intruded dikes, leading to ineligible density difference between the magma and encasing rocks, as well as overpressure increase from upward migration (Sigmundsson et al. 2024). The largest difference in parameter values between the Svartsengi and Krafla dike sequence model is the tapering factor, which is 2000 Pa/m for the Svartsengi case, 15 times larger than that for the Krafla case, possibly indicating varied stress along the oblique rift or dike propagating away from the preexisting weak zone (Jenness and Clifton 2009; Philippon et al. 2015; Greiner et al. 2025).

## Conclusions

To reexamine the role of tectonic stress and topography in controlling the spatial distribution of dike openings during the 1975–1984 Krafla rifting episode, north Iceland, we propose a revised version of the Buck et al. (2006) dike sequence model. In our model, a dike starts by lateral flow of magma from an inlet into a fissure swarm. The dike propagates along a positive gradient of driving pressure until the local maximum of driving pressure is reached. Magma intrusion stops once a pressure equilibrium between the dike and the inlet is re-established, that is, no pressure gradient to drive the dike further. The revised model (i) assumes a three-section dike geometry with tapering toward its far end, (ii) restricts the tectonic stress released by individual dike intrusions as well as by the entire dike sequence, (iii) incorporates magma compressibility in mass conservation between a magma domain and a set of dikes, and (iv) determines where dike tapering begins based on the cumulative stress profile before resolving the spatial distribution of dike opening. The model reproduces realistic dike geometries and magma pressure changes, in agreement with geophysical observations and geodetic models of rifting episodes, and our modeling provides deeper insights into the evolution of tectonic stress at divergent plate boundaries.

Using a plausible parameter setting, our model predicts dike openings and inlet overpressure variations that are consistent with the geophysical observations and previous models for the 1975–1984 Krafla rifting episode. The model indicates the fissure swarm inlet locates 2–4 km north of the Krafla caldera center. Parameter sensitivity analysis suggests a relatively low magma overpressure at the inlet of < 10 MPa prior to the first and the largest dike intrusion of the entire dike sequence, compared to > 20 MPa of initial dike driving pressure contributed by tectonic stress and topographic effects. Modeling results favor a failure pressure limit ratio of 2 between the first and successive dikes. A larger failure limit for the first dike compared to later dikes, a significant tectonic stress component along the dikes’ paths, and the compressibility of the magma domain permit magma flow into later dikes even when pressure conditions are below lithostatic at the LNB.

The model is adapted for the first three dikes in the 2023–2025 Svartsengi rifting episode, SW Iceland, during November 2023 to January 2024. Predicted dike openings have comparable magnitudes with those from geodetic inversions, and the predicted propagation directions of dikes causing eruptions are consistent with the locations of eruptive fissures. For the first dike, topographic effects contribute about 2 MPa higher driving pressure for the southward dike propagation than for the northward propagation. A buoyancy effect of 9 MPa and a tensile tectonic stress of 8 MPa allow the first dike to initiate when the overpressure from magma supply in the domain is 3 MPa lower than the lithostatic pressure. The model also indicates that the tensile stress at the plate boundary was at least 7.7 MPa and inhibited eruption during initial dike formation from the magma domain by preferentially channeling magma towards lateral propagation.

## Supplementary information

Below is the link to the electronic supplementary material.

ESM 1 (PDF 1.40 MB)

ESM 2 (MP4 17.0 MB)

ESM 3 (TXT 344 bytes)

## Data Availability

The study builds on published data.

## Code availability

The MATLAB scripts of the model are available upon request to the correspondence.
