# Supplementary material for: Role of tectonic stress and topography on repeated lateral dikes: application to the 1975–1984 Krafla and 2023–2025 Svartsengi rifting episodes in Iceland
Source: Bull Volcanol. 2025 Nov 4;87(12):105. doi: 10.1007/s00445-025-01897-y (PMC12586415; doi:10.1007/s00445-025-01897-y)
Supplement: Supplementary file 1 — (PDF 1.40 MB) [file 445_2025_1897_MOESM1_ESM.pdf]

Online resource 1: Supplementary text, figures and tables for model set-up and interpretation

Role of tectonic stress and topography on repeated lateral dikes: application to the 1975–1984 Krafla and 2023–2025 Svartsengi rifting episodes in Iceland

Yilin Yang[1] ✉ · Freysteinn Sigmundsson[1] · Halldór Geirsson[1] · Joachim Gottsmann[2]

[1] Nordic Volcanological Center, Institute and Faculty of Earth Sciences, University of Iceland, Reykjavík, Iceland

[2] Department of Earth and Environmental Sciences, Ludwig-Maximilians-Universität München, Munich, Germany

✉ **yiy1@hi.is**

This supplementary material provides additional information for the methodology, including: a list of symbols used in the manuscript (Table S1); the derivation of the relationship between dike opening and tectonic stress reduction for the model ('Derivation of Eq. (3) in the main text' and Fig. S1); the numerical expression of the segmented dike geometry for implementation ('Numerical expression of the segmented dike geometry'); and a demonstration of the searching in the numerical implementation of the model (Fig. S2). To further justify the model assumptions, a discussion on the connectivity between the inlet and rear section is also included. The driving pressure supplied by tectonic stress and topographic effects is referred to as external driving pressure in this text for simplicity.

To provide supplementary information for the model set-up for both applications, this material further includes the comparison between the smoothed and simplified topography along the Krafla fissure swarm (Fig. S3) and the model settings for the Svartsengi rifting episode (Table S2). Figure S4 compares the model predictions between the original and revised models. Figure S5 presents more modeling results for the Svartsengi rifting episode.

Table S1 List of symbols used in the manuscript in addition to model input parameters presented in Table 1. The positive or negative sign in the description column indicates the sign of the value of the parameters as typically used in the model. The last column shows where the parameters are involved; either influencing conditions in the dikes or in the fissure swarm inlet

Symbol	Description	Unit	Component
p_u	Magma overpressure gained during upward migration before lateral propagation (+)	Pa	Fissure swarm inlet
p_i	Magma overpressure at the fissure swarm inlet (+)	Pa	Fissure swarm inlet
Δp_i	Pressure change associated with magma extraction from the magma domain (-)	Pa	Fissure swarm inlet
p_e	Dike driving pressure supplied by topographic effects and tectonic stress (+)	Pa	Dike
$P_{driving}$	Net dike driving pressure including magma overpressure, buoyancy pressure and external contributions of p_e (+)	Pa	Fissure swarm inlet
$\sigma_{t,i}$	Tectonic stress at the fissure swarm inlet (varies with dike injections) (-)	Pa	Fissure swarm inlet
$\sigma_t(x)$	Tectonic stress at a location (x) along the dike path prior to dike intrusion (-)	Pa	Dike
$\Delta\sigma_t(x)$	Tectonic stress reduction at a location along the dike after dike intrusion (+)	Pa	Dike
ρ_d	Magma density in the dike (+)	kg/m^3	Dike
ρ_s	Magma density in the magma domain (+)	kg/m^3	Fissure swarm inlet
g	Gravity acceleration (+)	m/s^2	
$\Delta h(x)$	Surface elevation difference between a point along the dike path (location l) and the fissure swarm inlet (-)	m	Dike
$w(x)$	Dike opening at a location (x) along the dike path (+)	m	Dike
L	Length of the dike (+)	m	Dike
ΔL	Grid size of the dike length for numerical implementation (+)	m	Dike
d	Distance from the local maximum of driving pressure to a point in the front section of a dike (+)	m	Dike
A	A scaling factor used in the relationship between dike opening and tectonic stress reduction (+)	/	Dike
ΔM_d	Mass of a dike (after dike intrusion has ended) (+)	kg	Dike
ΔM_s	Mass change in the magma domain at the end of a dike intrusion (-)	kg	Fissure swarm inlet
β_s	Mechanical compressibility of the magma domain (depending on different assumptions on the magma domain geometry) (+)	Pa^{-1}	Fissure swarm inlet

Derivation of Eq. (3) in the main text

Equation 3 in the main text assumes that dike opening at each location along a dike scales with the release of pre-diking tectonic stress at that location. Qin and Buck (2008) suggested that the two-dimensional elastic model for an elliptical hole in an infinite region approximating the dike cross-section perpendicular to the dike plane (Fig. 2 in the main text) can provide a general guidance for calculating the dike opening. The dike opening (w) in this cross-section can be calculated by rewriting the equation (1B) and (4B) in Pollard et al. (1983) into:

$$w = \pm\frac{\Delta\sigma_e(1-\nu)\sqrt{\left(\frac{H}{2}\right)^2 - z^2}}{\mu}, \tag{S1}$$

where μ and ν are the shear modulus and Poisson's ratio of the host hock, respectively, H is the assumed height of the dike, z represents the vertical distance away from the central point of the dike cross-section, and $\Delta\sigma_e$ is the local tectonic stress change on the dike wall. The square root in Eq. (S1) diminishes to zero at the bottom and top of the dike. To derive the average opening on a dike cross-section, we integrate for Eq. (S1):

$$\int_{-\frac{H}{2}}^{\frac{H}{2}} w\mathrm{d}z = \frac{\Delta\sigma_e(1-\nu)}{\mu}\int_{-\frac{H}{2}}^{\frac{H}{2}}\left[\left(\frac{H}{2}\right)^2 - z^2\right]\mathrm{d}z = \frac{\Delta\sigma_e(1-\nu)}{\mu}\frac{\pi}{4}\left(\frac{H}{2}\right)^2, \tag{S2}$$

where only one side of the dike wall (positive part) is considered. The average of width of the dike is:

$$w_{avg} = \frac{4}{H}\int_{-\frac{H}{2}}^{\frac{H}{2}} w(x)\mathrm{d}z = \frac{\Delta\sigma_e(1-\nu)\pi H}{4\mu}. \tag{S3}$$

However, dikes forming in a rifting episode are highly influenced by their depths close to the free surface of the Earth, causing larger opening than anticipated in the full space approximation. A scaling factor, A, is introduced into Eq. (S3) and we have the Eq. (3) in the main text:

$$w(x) = A\frac{\pi H(1-\nu)}{4\mu}\Delta\sigma_t(x). \tag{S4}$$

To obtain an empirical value for this scaling factor A, we set up a finite element model (FEM) with uniform dike opening in a homogeneous elastic half space (Okada 1985; Pascal et al. 2014), using COMSOL Multiphysics® software v6.1 (2022). The dike is set to 3700 m height and 3000

m depth for the center, the same as estimated for the setting for the Krafla rifting episode. The elastic modulus of the host rock is also set the same as in the main text. We variate the length and the opening of the dike to investigate how the dike opening influence its surrounding stress field. The average normal stress on the dike plane at its central depth (Fig. S1b) within 2 km away from the dike is compared with the stress change derived with Eq. (S4) when $A = 2$ in Fig. S1a. The comparison indicates that for dike length larger than 10 km, normal stress change on the dike plane calculated using Eq. (S4) with $A = 2$ has only <10% difference from the FEM value. This empirical value is therefore taken in the derivation of the main text as the revised dike sequence model predicts dike lengths are in general larger than 10 km for the Krafla rifting episode, except for the ones with eruptions.

Numerical expression of the segmented dike geometry

In the main text, a dike is divided into three sections, the rear, central and front sections, extending from dike near end (nearest to the fissure swarm inlet) to the far end. The rear section features a uniform final external driving pressure. Within the central section, we apply uniform dike opening by assigning the maximum percentage of tectonic stress reduction $\Delta\sigma_t(x) = \alpha\sigma_{t,total}$. The front section starts from the location where dike driving pressure reaches its local maximum, and its opening tapers toward the dike far end.

If we denote a location along the dike as x, for x in the rear section, the external driving pressure (p_e) can be expressed numerically as follow:

$$p_e(x) = -\rho g\Delta h(x) - \sigma_t(x) - \Delta\sigma_t(x) = constant, \qquad \text{(S5)}$$

where $\Delta\sigma_t(x)$ is variable. The dike near end would overlap with the inlet when $-\rho g\Delta h(x) - \sigma_t(x) - \Delta\sigma_t(x) \leq -\sigma_{t,i}$, where $\sigma_{t,i}$ is the tectonic stress at the inlet. When $-\rho g\Delta h(x) - \sigma_t(x) - \Delta\sigma_t(x) > -\sigma_{t,i}$, the near end would be located where $\Delta\sigma_t(x) = 0$ (Fig. S2).

For x in the central section,

$$p_e(x) = -\rho g\Delta h(x) - \sigma_t(x) - \alpha\sigma_{t,total}. \qquad \text{(S6)}$$

Note that $\alpha\sigma_{t,total}$ is a constant for each dike. This section only exists when $p_e(x) < -(\rho g\Delta h_{max} + \sigma_{t,max}) - \alpha\sigma_{t,total}$ for x in the rear section, where the term $(\rho g\Delta h_{max} + \sigma_{t,max})$ represents the the local maximum value of the external driving pressure.

For x in the front section,

$$p_e(x) = -\rho g \Delta h_{max} - \sigma_{t,max} - \Delta\sigma_{t,max} + \varepsilon d - \rho g(\Delta h(x) - \Delta h_{max}), \qquad \text{(S7)}$$

where $\Delta\sigma_{t,max}$ represents the tectonic stress change at the local maximum of dike driving pressure, and d represents the distance further away from that location. Equation S7 implements a linear decay of tectonic stress relief further away from the local maximum of driving pressure, considering a tapering factor (ε) with distance (d) and a local topographic effect. Dike propagation is assumed to stop when

$$\varepsilon d = (1 - \gamma)\Delta\sigma_{t,max}, \qquad \text{(S8)}$$

or

$$p_e(x) = -\rho g \Delta h(x) - \sigma_t(x). \qquad \text{(S9)}$$

The first condition is usually for positive to minor negative gradients of initial driving pressure in the front section, while the second is for significant negative gradient of initial driving pressure. The far end locates in an area with zero or negative gradient of driving pressure, otherwise the dike will propagate further to next area where driving pressure reaches its local maximum.

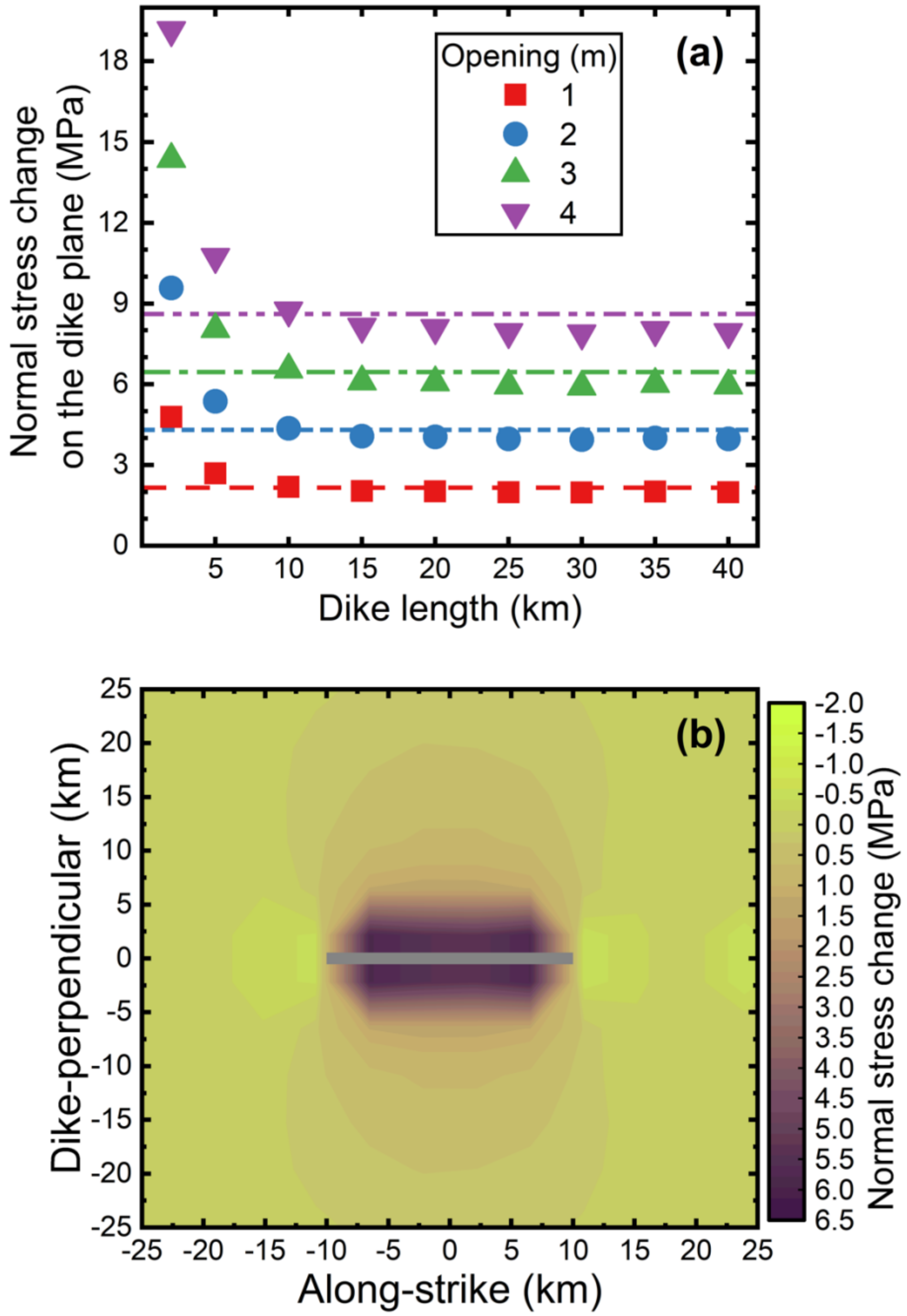

Fig. S1 Stress change induced by a uniform dike opening in a uniform elastic half-space. **a** The relationship between the dike length, dike opening and normal stress change on the dike plane. The dashed lines are calculated with Eq. (S4) and a scaling factor $A = 2$. **b** Exemplified spatial distribution of normal stress change on the dike plane at central depth of the dike, 3000 m. The grey line indicates the location of the dike. The dike has a 20 km length and opens 3 m, corresponding to the middle green triangle in Fig. S1a

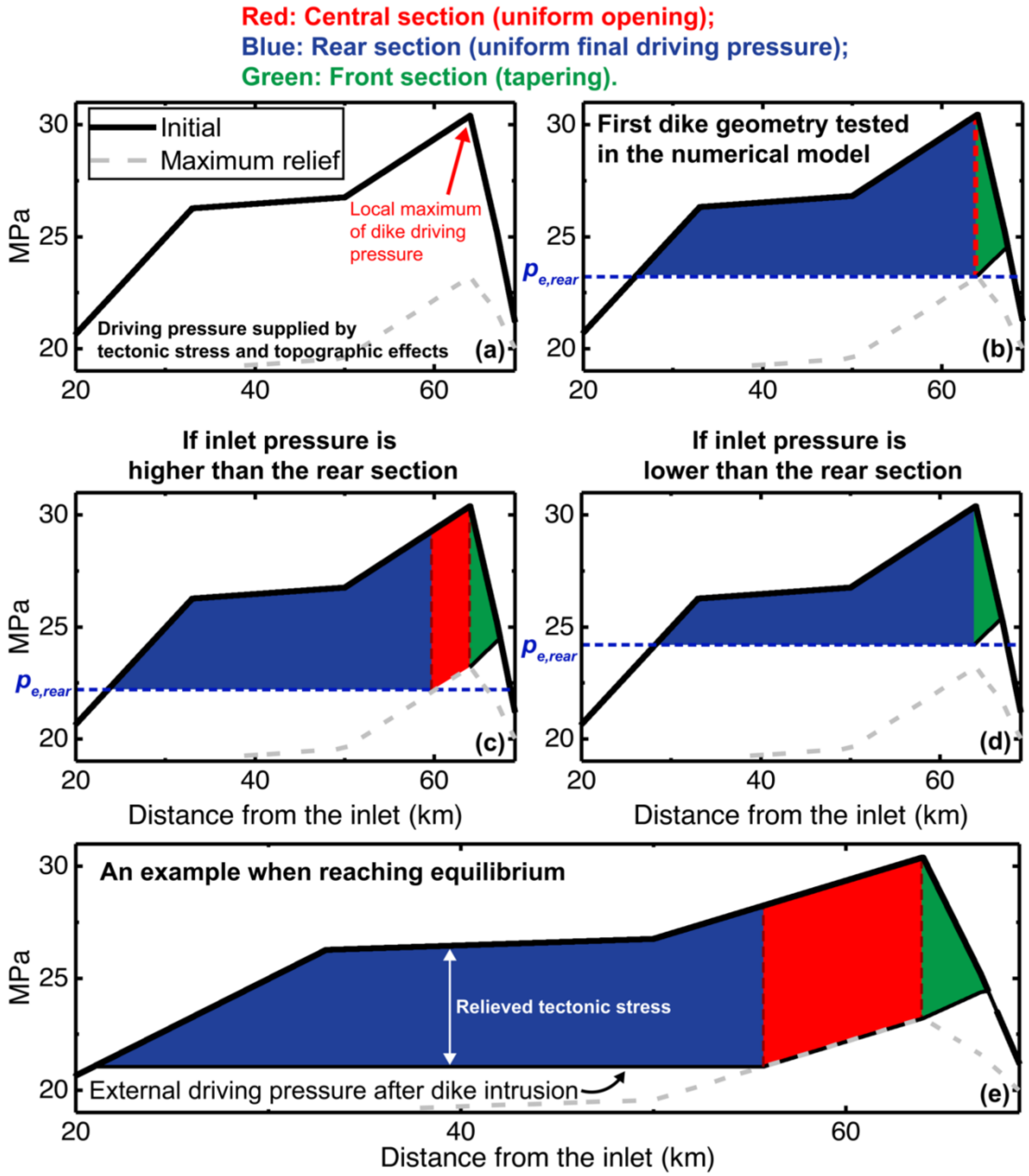

Fig. S2 Demonstration of our numerical modeling scheme and dike geometry indicated by external driving pressure distribution, exemplified with the first diking event of the Krafla rifting episode. **a** Initial external driving pressure. The grey dashed line indicates the external driving pressure level after the maximum percentage of tectonic stress is relieved. **b** The dike geometry first tested in the procedure of numerical modeling, of which the dike only has maximum opening at the local maximum of dike driving pressure (red vertical dashed line). The front section is determined from the local maximum of driving pressure and the tapering parameters. **c** and **d** show how the numerical model continues searching for the final pressure at the inlet and in the rear section. Panel c shows the case when the overpressure at the inlet is higher than the rear section after considering a pressure drop caused by a dike in panel b. Panel d shows the case when the overpressure at the inlet is lower than the rear section after considering a pressure drop caused by a dike in panel b. **e** An example of final external driving pressure when the equilibrium of the inlet-dike system is re-established

Connectivity between the inlet and rear section

Figure 4b in the main text shows that the openings of the first, second and sixth dikes locate offset laterally from the fissure swarm inlet. In this case the magma must travel in the fissure swarm and maintain the connection to the inlet for magma supply. Rivalta (2010) assumed a cylinder conduit with radius of 1 m connecting dike(s) and the feeding source to predict the flow rate variation during a dike intrusion. Tryggvason (1984) assigned ~0.5 m of surface widening between the inlet and the area with widening larger than 1 m. This connectivity is needed to predict the detailed dynamic processes of magma flow, but considered to have a negligible effect on our models for predicting the first-order regularities of a dike sequence.

The connectivity has limited influence on the eventual manifestation of a dike sequence. The magma volume related to this connectivity is small. Assuming the connectivity as a dike with opening of 0.5 m, height of 3700 m and length of 20 km for the first event in Fig. 4b in the main text, the magma volume within is only ~12% of the magma volume for the major dike opening. For the second and sixth dikes, this percentage decreases to ~10% and <6%, respectively. The conduit connectivity assumption in Rivalta (2010) yields smaller magma volume.

How the magma in this connectivity interact with later intrusions is uncertain. Despite that tectonic stress will be released if magma flow through the conduit deforms the host rock, reducing the tectonic stress and providing less driving pressure for later events, these magma conduits may also generate a zone of weakness in the crust supporting magma flow (Jenness and Clifton 2009). The general underestimation of volume loss in the magma domain during the latter two sub-sequences of dikes in Fig. 4e of the main text may demonstrate the complexity of these processes. Considering the connectivity in a detailed manner for this type of dike model we present requires more assumptions than we invoke here, and eventually causes problems in the model implementation.

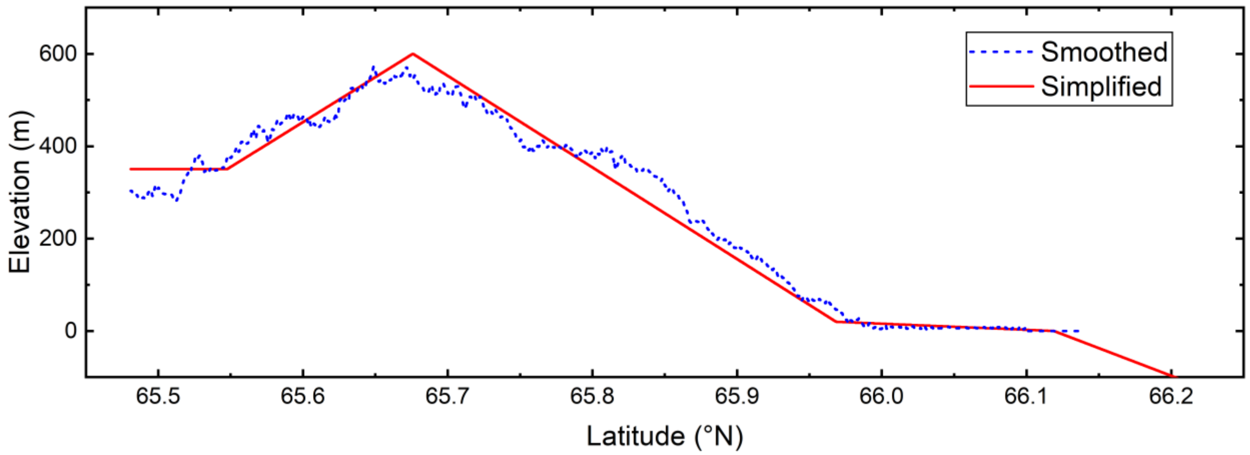

Fig. S3 Comparison between the elevation profile smoothed from actual topography and simplified for the revised dike sequence model. The blue curve is the same as shown in Fig. 1c in the main text

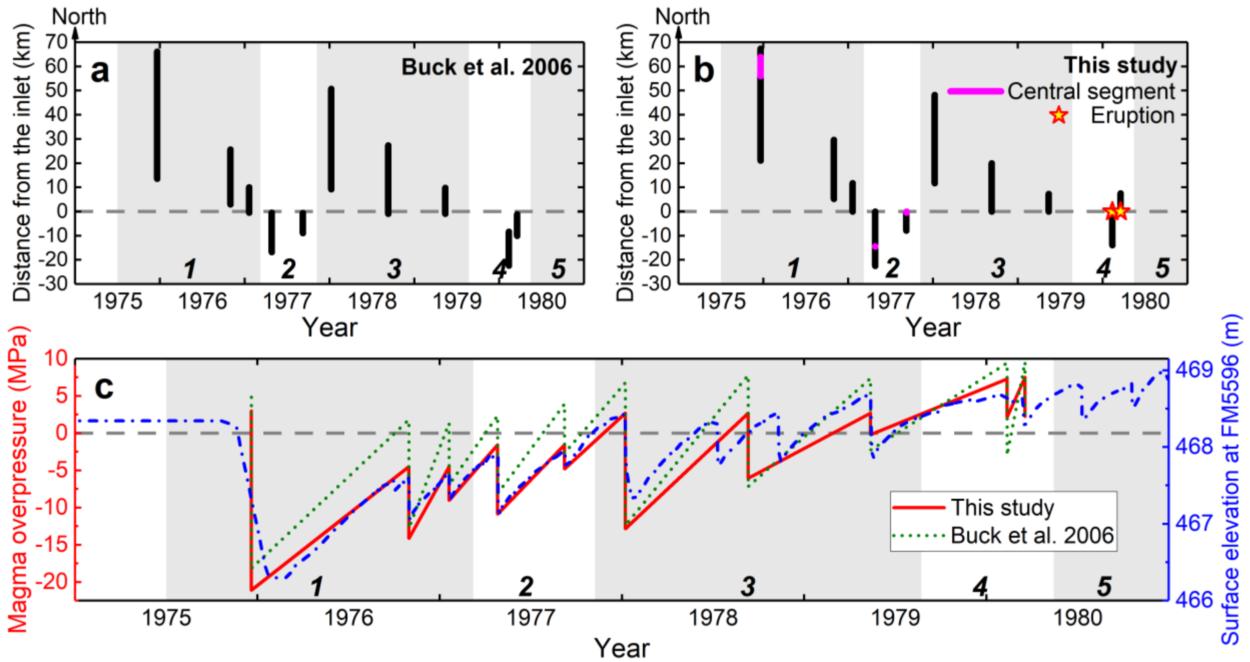

Fig. S4 Comparison between model prediction by the models in Buck et al. (2006) and this study. **a** The spatial distribution of dike openings predicted by the original model, extracted from Fig. 11 in Buck et al. (2006). **b** The spatial distribution of dike openings predicted by the revised model **c** Variation of the magma overpressure at the inlet predicted by the models, compared with the surface elevation change in the Krafla caldera center. The green curve is from Fig. 11 in Buck et al. (2006)

Table S2 A plausible setting of parameters of the revised dike sequence model for the 2023–2025 Svartsengi rifting episodes.

Parameter		Value	Reference
Tectonic stress $\sigma_{t,total}$ [MPa]		-8.0	Sigmundsson et al. (2024)
Pressure added from upward migration of magma before its lateral propagation (p_u) [MPa]		6.0	Sigmundsson et al. (2024)
Tensile strength P_o	First dike [MPa]	20.0	Parks et al. (2025)
	Rest of dikes [MPa]	5.0	
Magma domain	Radius R [m]	2510	Sigmundsson et al. (2024)
	Compressibility β_m [Pa^{-1}]	5.30×10^{-11}	Sigmundsson et al. (2024)
Host rock (<5 km depth)	Density ρ [kg/m^3]	2510	Table 1
	Poisson's ratio ν	0.20	Sigmundsson et al. (2024)
Dike	Height H [m]	3200	Sigmundsson et al. (2024)
	Central depth [m]	3000	Sigmundsson et al. (2024)
Maximum percentage of tectonic stress relieved by one dike α		80%	
Stress reduction ratio between the far end and the local maximum of dike driving pressure γ		0.30	
Tapering factor ε [Pa/m]		2000	

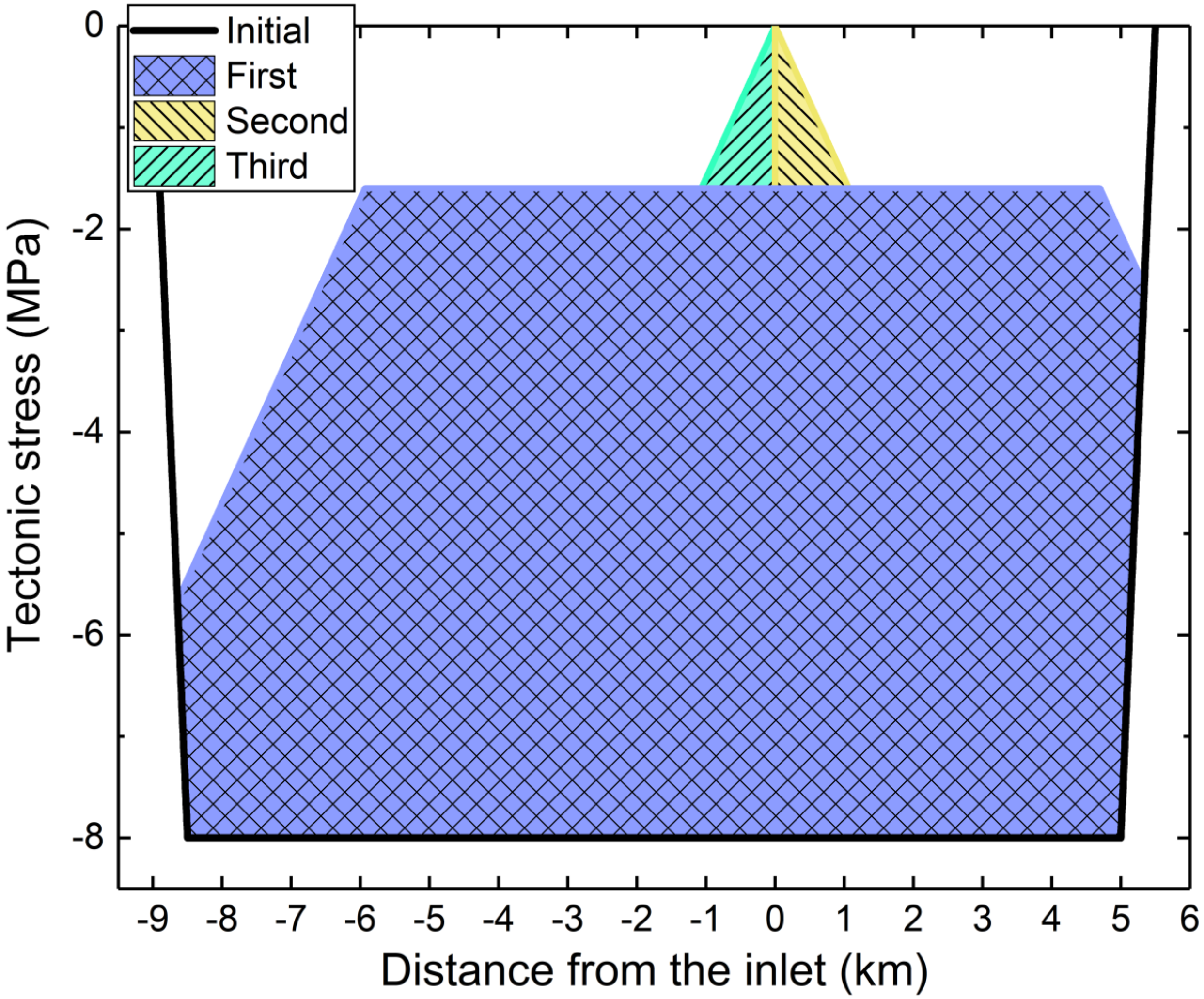

Fig. S5 Variation in tectonic stress predicted by the revised dike sequence model for the first three dike intrusions at the Sundhnúkur crater row. The tensile tectonic stress accumulated prior to the rifting episode is assumed to be 8 MPa with stress barriers on both sides away from the inlet.

References in this supplementary material

COMSOL Multiphysics® (2022) version 6.2. COMSOL AB, Stockholm, Sweden.

Buck WR, Einarsson P, Brandsdóttir B (2006) Tectonic stress and magma chamber size as controls on dike propagation: Constraints from the 1975–1984 Krafla rifting episode. J Geophys Res Solid Earth 111:B12404. https://doi.org/10.1029/2005JB003879

Greiner SHM, Sigmundsson F, Geirsson H, Burchardt S, Galland O (2025) Tectonic stress as the driving mechanism for dike opening in an oblique rift setting: A deformation model of the 2021 Fagradalsfjall dike, Iceland. Geophys Res Lett 52: e2024GL113970. https://doi.org/10.1029/2024GL113970

Jenness MH, Clifton AE (2009) Controls on the geometry of a Holocene crater row: a field study from southwest Iceland. Bull Volcanol 71:715-728. https://doi.org/10.1007/s00445-009-0267-9

Okada Y (1985) Surface Deformation due to Shear and Tensile Faults in a Half-Space. Bulletin of the Seismological Society of America 75:1135-1154. https://doi.org/10.1785/BSSA0750041135

Parks M, Drouin V, Sigmundsson F, Hjartardóttir ÁR, Geirsson H, Pedersen GBM et al (2025) 2023–2024 inflation-deflation cycles at Svartsengi and repeated dike injections and eruptions at the Sundhnúkur crater row, Reykjanes Peninsula, Iceland. Earth Planet Sci Lett 658:119324. https://doi.org/10.1016/j.epsl.2025.119324

Pascal K, Neuberg J, Rivalta E (2014) On precisely modelling surface deformation due to interacting magma chambers and dykes. Geophys J Int 196(1):253-278. https://doi.org/10.1093/gji/ggt343

Pollard DD, Delaney PT, Duffield WA, Endo ET, Okamura AT (1983) Surface Deformation in Volcanic Rift Zones. Developments in Geotectonics 19:541-559. https://doi.org/10.1016/B978-0-444-42198-2.50036-5

Qin R, Buck WR (2008) Why meter-wide dikes at oceanic spreading centers? Earth Planet Sci Lett 265:466-474. https://doi.org/10.1016/j.epsl.2007.10.044

Rivalta E (2010) Evidence that coupling to magma chambers controls the volume history and velocity of laterally propagating intrusions. J Geophys Res Solid Earth 115:B07203. https://doi.org/10.1029/2009JB006922

Tryggvason E (1984) Widening of the Krafla Fissure Swarm during the 1975-1981 Volcano-Tectonic Episode. Bull Volcanol 47:47-69. https://doi.org/10.1007/BF01960540

Parks M, Drouin V, Sigmundsson F, Hjartardóttir ÁR, Geirsson H, Pedersen GBM et al (2025) 2023–2024 inflation-deflation cycles at Svartsengi and repeated dike injections and eruptions at the Sundhnúkur crater row, Reykjanes Peninsula, Iceland. Earth Planet Sci Lett 658:119324. https://doi.org/10.1016/j.epsl.2025.119324

Sigmundsson F, Parks M, Geirsson H, Hooper A, Drouin V, Vogfjörd KS, et al. (2024) Fracturing and tectonic stress drive ultrarapid magma flow into dikes. Science 383(6688):1228-1235. https://doi.org/10.1126/science.adn2838
